# Tanreqing Inhibits LPS-Induced Acute Lung Injury *In Vivo* and *In Vitro* Through Downregulating STING Signaling Pathway

**DOI:** 10.3389/fphar.2021.746964

**Published:** 2021-10-14

**Authors:** Yu-Qiong He, Can-Can Zhou, Jiu-Ling Deng, Liang Wang, Wan-Sheng Chen

**Affiliations:** ^1^ Institute of Chinese Materia Madica, Shanghai University of Traditional Chinese Medicine, Shanghai, China; ^2^ Department of Pharmacy, Changzheng Hospital, Second Military Medical University, Shanghai, China; ^3^ Department of Pharmacy, Shanghai Tenth People’s Hospital, Tongji University School of Medicine, Shanghai, China; ^4^ Suzhou Chien-Shiung Institute of Technology, Taicang, China

**Keywords:** tanreqing injection, acute lung injury, STING, inflammation, oxidative stress

## Abstract

Acute lung injury (ALI) is a common life-threatening lung disease, which is mostly associated with severe inflammatory responses and oxidative stress. Tanreqing injection (TRQ), a Chinese patent medicine, is clinically used for respiratory-related diseases. However, the effects and action mechanism of TRQ on ALI are still unclear. Recently, STING as a cytoplasmic DNA sensor has been found to be related to the progress of ALI. Here, we showed that TRQ significantly inhibited LPS-induced lung histological change, lung edema, and inflammatory cell infiltration. Moreover, TRQ markedly reduced inflammatory mediators release (TNF-α, IL-6, IL-1β, and IFN-β). Furthermore, TRQ also alleviated oxidative stress, manifested by increased SOD and GSH activities and decreased 4-HNE, MDA, LDH, and ROS activities. In addition, we further found that TRQ significantly prevented cGAS, STING, P-TBK, P-P65, P-IRF3, and P-IκBα expression in ALI mice. And we also confirmed that TRQ could inhibit mtDNA release and suppress signaling pathway mediated by STING *in vitro*. Importantly, the addition of STING agonist DMXAA dramatically abolished the protective effects of TRQ. Taken together, this study indicated that TRQ alleviated LPS-induced ALI and inhibited inflammatory responses and oxidative stress through STING signaling pathway.

## Introduction

Acute lung injury (ALI) and its more serious manifestation-acute respiratory distress syndrome (ARDS) are respiratory diseases characterized by acute hypoxemic respiratory distress and severe lung edema with normal cardiac filling pressures (Yang W. et al., 2020). Pneumonia, sepsis, aspiration, and trauma are the major causes of ALI/ARDS (Suresh et al., 2000). Despite improvements in therapy methods over the years, the mortality rate remains high. A prospective study conducted in 14 Brazilian ICU containing 7,133 patients showed that the mortality rate associated with ALI reached 49.2% (Caser et al., 2014). Moreover, a study conducted in Japan also indicated that the mortality of patients with ARDS was 38% (Fujishima et al., 2020). Therefore, it is urgent to understand the pathophysiology of ALI/ARDS and find effective treatment.

Tanreqing injection (TRQ), a well-known Chinese patent medicine, is approved by the National Drug Regulatory Authority of China (No: Z20030045) for the treatment of phlegm-heat lung obstruction (Huang et al., 2019). TRQ is extracted and processed from five ingredients, namely, *Scutellaria baicalensis* Georgi. [Lamiaceae; Scutellariae radix], *Selenaretos thibetanus* Cuvier [Ursidae; Ursi Fellis Pulvis], *Capra hircus* Linnaeus [Bovidae; Caprae Hircus Cornu], *Lonicera japonica* Thunb. [Caprifoliaceae; Lonicerae Japonicae Flos], and *Forsythia suspensa* (Thunb.) Vahl [Oleaceae; Forsythiae Fructus] (Rivera et al., 2014). We have previously reported that TRQ consists of at least 107 compounds, including flavonoids, phenolic acids, lignins, steroids, phenylethanoid glycosides, and other components (Wang L. et al., 2021). The pharmacological experiments have shown that TRQ has excellent antibacterial *in vitro* and anti-inflammatory activities in mucus hypersecretion models (Wang et al., 2011; Liu et al., 2020). Clinical studies have revealed that TRQ shows therapeutic effects in respiratory diseases such as pneumonia, acute bronchitis, and acute exacerbation of chronic obstructive pulmonary disease (AECOPD) (Yang Y. et al., 2020). More importantly, TRQ has been recommended by numerous guidelines and expert consensus for the treatment of severe and critically ill patients with COVID-19 (Zhuang et al., 2020). However, the effect and mechanism of TRQ on ALI remain elusive.

STING (also refereed to Tmem173) as a cytosolic DNA sensor can promote type I interferon (IFN) expression during innate immune signaling (Hou et al., 2018). An increasing number of investigations have shown that STING is involved in various diseases; the activation of STING can develop as cancer therapeutics. However, the activation of STING can also induce inflammatory responses and lead to inflammatory injury (Amouzegar et al., 2021; Decout et al., 2021). The activation of STING is associated with inflammatory diseases such as acute pancreatitis, sepsis, and rheumatoid arthritis (Jeremiah et al., 2014; Heipertz et al., 2017; Zhao et al., 2018; Wang et al., 2019). The deficiency of STING could alleviate intestinal ischemia-induced kidney, lung, and liver injury, which might be related to the reduction of inflammatory responses and oxidative stress (Wu et al., 2021). Intriguingly, STING was found to be largely associated with the process of virus infection. In the early stage of SARS-CoV-2 infection, the STING signaling was inhibited. Then the STING will be excessively activated by the damaged self-DNA, causing mass release of inflammatory mediators and a cytokine storm (Berthelot et al., 2020; Berthelot and Liote, 2020). And loss of STING function in bats leads to weakened immune-inflammatory responses, resulting in the coexist of bats with various viruses (Xie et al., 2018). Meanwhile, Li et al. showed that the STING pathway was activated in LPS-induced ALI mice and in LPS-stimulated macrophages, and the deficiency of STING could alleviate LPS-induced lung injury in mice (Ning et al., 2020). Therefore, drugs blocking STING pathway may effectively prevent ALI.

We hypothesis that TRQ could interfere STING signaling pathway, prevent excessive inflammatory responses and oxidative stress, and then suppress ALI. Our study might provide new insight into TRQ for the treatment of respiratory-related diseases.

## Materials and Methods

### Regents and Chemical

Tanreqing injection (TRQ, 33 mg/ml) was provided by Shanghai Kaibao Pharmaceutical Company, China, Lot. No. 2003210. LPS (*Escherichia coli* 055: B5, L2880) was purchased from Sigma-Aldrich (St. Louis, MO, United States). Fetal bovine serum (FBS) was obtained from Invitrogen Gibco (Grand Island, NY). Penicillin and streptomycin, Dulbecco’s modified Eagle’s medium (DMEM), 0.25% trypsin, and phosphate buffer saline (PBS) were purchased from Meilunbio (Dalian, China). Antibodies against cGAS, P-STING, STING, P-TBK, TBK, P-IRF3, IRF3, NF-κB p65, P-P65, and P-IκBα were obtained from Cell Signaling Technology (Beverly, MA, United States). All the immunosorbent assay (ELISA) used in this study were acquired from Multiscience (Zhejiang, China), Elabscience (Wuhan, China), and Neobioscience Technology Company (Shenzhen, China). The MPO, LDH, MDA, GSH, and SOD assay kits were purchased from Nanjing Jiancheng Biology Institution (Nanjing, China). DNeasy Blood & Tissue Kit was purchased from Qiagen (Hilden, Germany). TB Green Premix Ex Taq II was acquired from TaKaRa (Beijing, China).

### HPLC-UV-ELSD Analysis of the Chemical Compounds of TRQ

The main chemical compounds of TRQ were detected on a Diamonsil C18 column (4.6 mm × 250 mm, 5 μm, Agilent Technologies, Santa Clara, CA, United States) conducted on an Agilent 1260 Series HPLC system. The detection was accomplished by DAD detector (254 nm) and ELSD detector (drift tube temperature: 95°C, atomization temperature: 60°C, and carrier gas flow rate: 1.60 ml/min). The column temperature was 30°C. Solvent A is acetonitrile; solvent B is 0.3% formic acid, 0–6 min, 5–27% A; 6–10 min, 27% A; 10–25 min, 27–54% A; 25–33 min, 54–85% A; 33–35 min, 85% A. Data were collected and proceeded by ChemStation 10.2.

### Animals

C57BL/6 mice (male, 6–8 weeks of age) were purchased from Cavens Company (Changzhou, Jiangsu, China) (Certificate No: SCXK (Su) 2018–0002). This animal protocol and procedure were in accordance with the National Institutes of Health guidelines for animal care and approved by the Ethics Committee for Animals of the Second Military Medical University (Approved No: 201802422). The mice were kept in cages with free access to water and food.

### Murine Model of ALI

The mice were randomly divided into six groups: Control, LPS, LPS + TRQL, LPS + TRQH, LPS + DEX, and LPS + TRQH + DMXAA. The doses of TRQ were converted from the clinical use of TRQ and referenced to the previous study (Fan et al., 2014). Briefly, the mice in the LPS + TRQH + DMXAA group were pretreated with DMXAA (ip, 10 mg/kg) for 1 h before the treatment of TRQ (Zhao et al., 2018). The mice in the LPS + TRQL, LPS + TRQH, LPS + DEX, and LPS + TRQH + DMXAA groups separately received TRQ (2.6 ml/kg), TRQ (5.2 ml/kg), DEX (1 mg/kg), and TRQ (5.2 ml/kg) by intraperitoneal (ip) injection; 1 h later, the mice in the last five groups were ip injected with LPS (5 mg/kg) dissolved in saline, while the mice in the Control group were ip injected with saline. After LPS administration for 6 h, the mice were sacrificed. Subsequently, the serum, bronchoalveolar lavage fluid (BALF), and lung tissues were collected for further test.

### Bronchoalveolar Lavage Fluid Collection and Analysis

The method for BALF collection has been described previously (Zhao et al., 2021). In brief, the mice were sacrificed and the thoracic cavity was opened, then the left lung bronchus was ligated, and 2 ml PBS was injected into trachea and withdrawn three times. And the BALF samples were centrifuged at 2000 rpm for 10 min at 4°C. The supernatant was collected and its protein concentrations were detected by a BCA protein assay kit. At the same time, the cells were lysed by ACK Lysis Buffer, washed with 1×PBS, and resuspended in 50 μL PBS. Finally, Wright-Giemsa staining was conducted, and the total cells and neutrophils were counted with a hemocytometer.

### Lung Wet/Dry (W/D) Ratios

The lung tissues were collected, weighed, and placed in an incubator at 60°C for 48 h to obtain the dry weight. Then, the ratio of wet lung weight to dry lung weight was calculated to measure lung edema.

### Histopathological Evaluation

The lungs of mice without BALF collection were fixed in 4% paraformaldehyde, dehydrated with a series of ethanol, embedded in paraffin, and cut into 5 μm thick sections. Then, the lung sections were stained with hematoxylin and eosin (H&E). Next, the lung tissues injury scores were obtained through elevating the degree of neutrophils infiltration in alveolar spaces or interstitial spaces, hyaline membranes formation, proteinaceous debris filling the airspaces, and alveolar wall thickening (Matute-Bello et al., 2011). And the grading of lung injury was calculated from 0 (normal) to 5 (severe) according to the pathological categories.

### Immunohistochemistry Analysis

The lung tissues section embedded in paraffin was deparaffinized with xylene, rehydrated with gradient ethanol, and quenched with 3% H_2_O_2_. After incubating with 1% bovine serum albumin (BSA) for blocking nonspecific protein, the sections were incubated with primary antibody overnight at 4°C. Next, the sections were incubated with secondary antibody, dyed with diaminobenzidine, and finally observed with a fluorescence microscope.

### Measurement of MPO, LDH, MDA, GSH, and SOD Content

The lung tissues were homogenized with homogenizer for the analysis of MPO, LDH, MDA, SOD, and GSH levels. The MPO, LDH, MDA, SOD, and GSH assay kits were purchased from Nanjing Jiancheng Biology Institution (Nanjing, China) and used following the manufacturer’s instructions.

### Cell Culture and Cell Viability

The RAW 264.7 cells (a macrophage line of mouse) were bought from the Cell Bank of the Chinese Academy of Sciences, Shanghai, China. Cells were cultured in DMEM supplemented with 10% [v/v] fetal bovine serum (FBS), 100 U/mL penicillin, and 100 μg/ml streptomycin at 37°C in a humidified atmosphere containing 5% CO_2_. The mouse bone marrow neutrophils were isolated from the femurs of C57BL/6 male mice (6–8 weeks) as previously described (Li et al., 2020). Briefly, the bone marrow from the femurs was filtered over a 70 μm nylon cell strainer and washed with PBS, and the red blood cells were lysed. Then percoll solution (55, 65, and 78%) was used to isolate neutrophils. And the neutrophils at the interface of the 65% percoll and 78% percoll were collected, washed, and resuspended in RPMI 1640 supplemented with 10% FBS, 100 U/mL penicillin, and 100 μg/ml streptomycin. Neutrophils were stained with Wright-Giemsa to determine their purity.

Cell viability was detected by using CCK-8 agents according to the manufacturer’s instructions. RAW 264.7 cells were seeded at 1×10^5^ cells per well in 96-well plates and incubated overnight. Then, the cells were treated with different concentrations of TRQ (0, 0.5, 1, 5, 10, 50, 100, or 200 μg/ml) for another 24 h. At the end of incubation, 10 μl CCK-8 agents were added to every well, which were incubated for another 1 h. Ultimately, the absorbance was detected at 450 nm.

### ELISA Assay

The serum of mice was collected and used to measure TNF-α, IL-6, IL-1β, and IFN-β levels with ELISA kits following the manufacturer’s instruction. Furthermore, RAW 264.7 cells were seeded into 48-well plates and cultured overnight. Then, DMXAA (10 μg/ml) and TRQ (10, 20, or 40 μg/ml) with or without LPS (1 μg/ml) were added to each well. 24 h later, the supernatants were harvested to measure the NO, TNF-α, IL-6, IL-1β, and IFN-β secretion by using ELISA kits.

### ROS Staining

The expression of ROS in cells was detected by the Reactive Oxygen Species Assay Kit (Beyotime, Shanghai, China). Briefly, cells were incubated with 10 μM DCFH-DA diluted in DMEM for 20 min at 37°C. At the end of incubation, cells were washed with DMEM three times and observed with fluorescent microscopy (Olympus, Japan).

### Quantitative Real-Time PCR Analysis

The extract and measurement of mtDNA were conducted as previously described (Bronner and O'Riordan, 2016). Briefly, cells were washed with PBS and lysated with 1% NP-40 on ice. The lysates were certificated at 13,000 rpm for 15 min at 4°C, and the supernatant was collected to extract mtDNA following the instructions of DNeasy Blood &Tissues Kit. Finally, qRT-PCR was performed using TB Green Premix Ex Taq II. The content of mtDNA was detected by measuring mitochondrially encoded genes (*mt-Co1*, *mt-Nd6*, and *mt-Cytb*), while *18S rDNA* was used as internal control. The 2^−ΔΔCt^ method was used to calculate the relative expression of targeting genes. The PCR primers used are shown in Supplementary Table S1.

### Immune-Fluorescent Staining

Cells were washed with PBS, fixed with 4% paraformaldehyde for 15 min, permeabilizated with 0.1% Triton-100 for 10 min, and then blocked with 3% BSA in PBS for 2 h. Subsequently, cells were incubated with p65 antibody at 4°C overnight, incubated with Alexa Fluor 488 for 1 h, and then stained with DAPI for 5 min at room temperature. Finally, the images were photographed by fluorescent microscopy (Olympus, Japan).

### Western Blot

The lung tissues of mice and RAW 264.7 cells were homogenized in a RIPA buffer to extract protein. BCA protein kit was used to measure protein concentration. Then, the proteins were separated with a 10% SDS-PAGE gel and transferred to a PVDF membrane. Next, the membrane was blocked with 5% BSA in TBST and incubated with primary antibodies at 4°C overnight. The following day, the membrane was washed with TBST three times and then incubated with secondary antibodies for 1 h at room temperature. Finally, the membrane was washed with TBST three times, visualized with electrochemiluminescence (ECL) reagent (Tanon, Shanghai, China), and measured by ChemiScope Imager (Clinx, Shanghai, China).

### Statistical Analysis

All data in this study were analyzed with the GraphPad Prism 5.0 statistical software. Data were presented as mean ± SEM. Shapiro–Wilk test to determine that whether the data were normally distributed. The nonparametric data were analyzed by the Mann–Whitney test or Kruskal–Wallis followed by Dunn’s post hoc test. Parametric data were analyzed by one-way ANOVA followed by Dunnett’s multiple comparisons test. The difference was considered significant when *p*-value < 0.05.

## Results

### Analysis of the Chemical Compounds of TRQ

**FIGURE 1:**
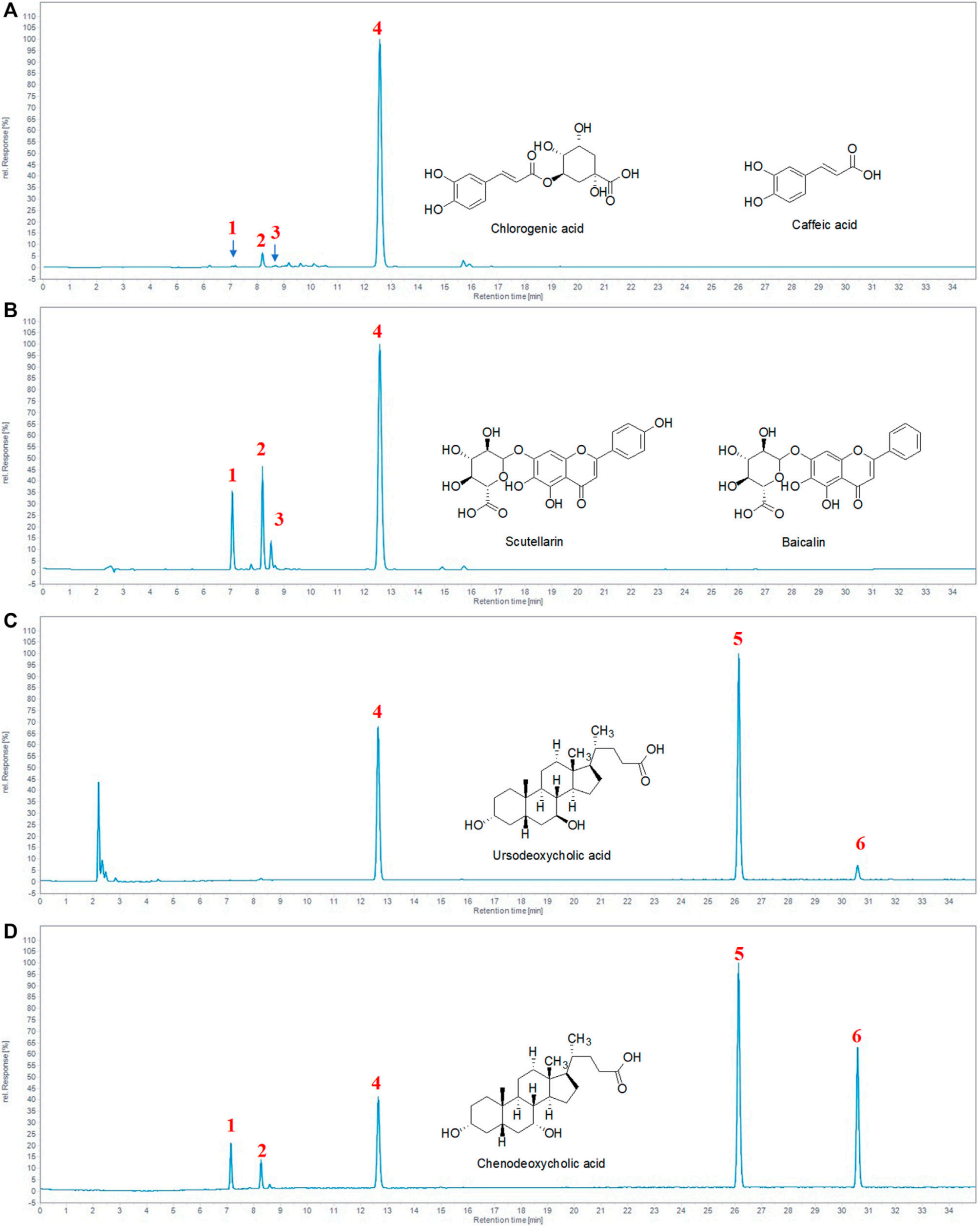
Identification of the major chemical components of TRQ. **(A–B)** The UV chromatogram of TRQ and mixed standards. **(C–D)** The ELSD chromatogram of TRQ and mixed standards. The numbers 1, 2, 3, 4, 5, and 6, respectively, represent chlorogenic acid, caffeic acid, scutellarin, baicalin, ursodeoxycholic acid, and chenodeoxycholic acid.

We performed HPLC to identify the chemical constituents of TRQ and analyzed the six main peaks of TRQ by comparing the retention time with the standards (Figures 1A–D). The quantitative analysis revealed that the contents of chlorogenic acid, caffeic acid, scutellarin, baicalin, ursodeoxycholic acid, and chenodeoxycholic acid were 0.05 mg/ml, 0.18 mg/ml, 0.06 mg/ml, 6.63 mg/ml, 4.79 mg/ml, and 0.67 mg/ml, respectively.

### TRQ Alleviated LPS-Induced Lung Injury in Mice

**FIGURE 2:**
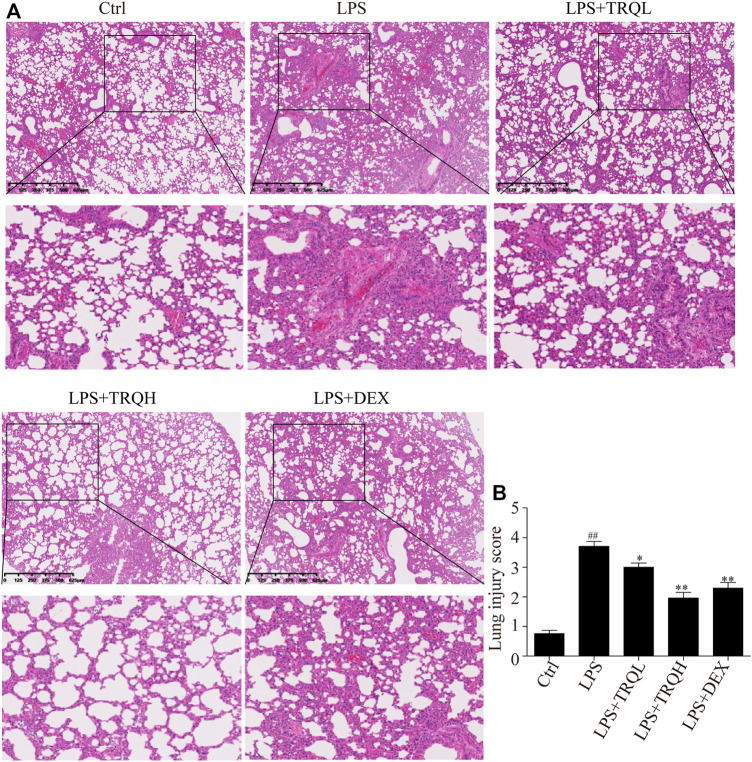
TRQ alleviated lung histopathological changes in LPS-induced ALI mice. **(A)** Representative H&E staining of lung tissues. **(B)** Quantification of lung injury. *n* = 5. ^*##*^ *p* < 0.01 vs. Ctrl group; ^*^ *p* < 0.05 and ^**^ *p* < 0.01 vs. LPS group.

In order to investigate the lung histopathological changes of mice, we stained the lung tissues with H&E staining and then observed them under light microscope. There were thickened alveolar wall and obvious inflammatory cells accumulation in the alveolar cavity in the lung tissues of mice treated with LPS (Figure 2A). However, these histopathological changes induced by LPS were significantly suppressed by TRQ or DEX pretreatment. In addition, the lung injury score was measured according to the degree of lung injury. LPS treatment caused a high lung injury score, which was markedly decreased by TRQ or DEX pretreatment (Figure 2B). These results indicated that TRQ could prevent the progress of ALI.

### TRQ Reduced Lung Edema and Cell Infiltration in ALI Mice

**FIGURE 3:**
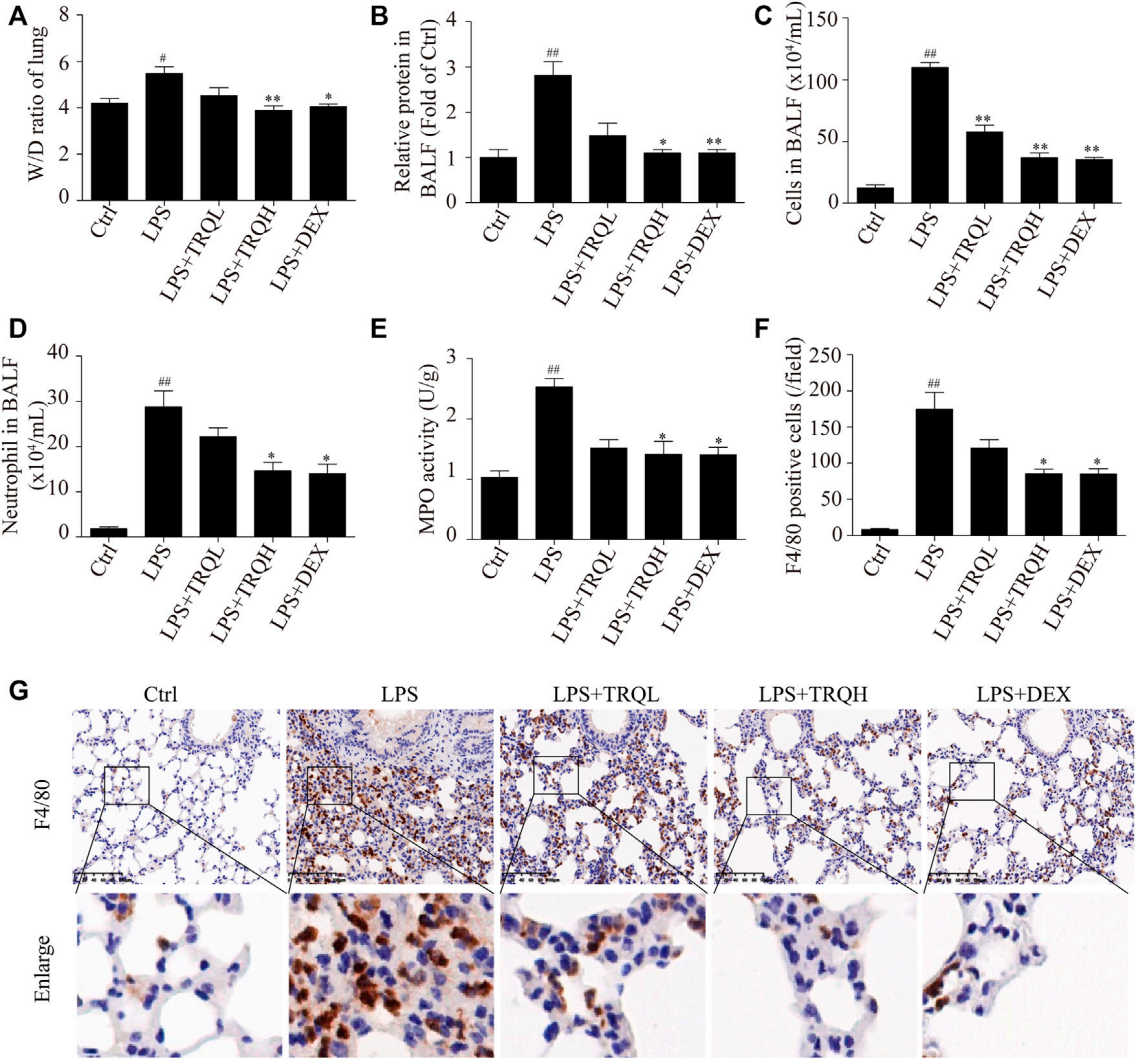
TRQ alleviated lung edema and inflammatory cells infiltration in LPS-induced ALI mice. **(A)** Lung W/D ratio. **(B)** Total protein concentration in BALF. **(C)** Total cell number in the BALF. **(D)** The number of neutrophils in BALF. **(E)** MPO activity in lung injury. **(F–G)** Representative images and quantitative analysis of macrophages stained with the macrophage marker protein F4/80. *n* = 5–6. ^*#*^ *p* < 0.05 and ^*##*^ *p* < 0.01 vs. Ctrl group; ^*^ *p* < 0.05 and ^**^ *p* < 0.01 vs. LPS group, respectively.

To investigate the effects of TRQ on lung edema, we measured lung W/D ratio and total protein in BALF. Compared to the Ctrl group, LPS treatment obviously caused a higher lung W/D ratio and protein content. At the same time, the pretreatment of TRQ or DEX obviously improved LPS-induced lung edema by decreasing lung W/D ratio and total protein in BALF (Figures 3A,B). Furthermore, the total cells and neutrophils in BALF were counted. TRQ significantly reduced the total cell number and neutrophils increased by LPS in BALF (Figures 3C,D). In addition, TRQ also inhibited the LPS-elevated lung MPO activity, which was considered as an indicator of neutrophil accumulation in the lung (Figure 3E). What is more, the F4/80 staining indicated that LPS also increased macrophage infiltration in lung tissues, while TRQ treatment decreased the macrophage infiltration (Figures 3F,G). Taken together, these data showed that TRQ could prevent lung edema and inflammatory cells infiltration in LPS-induced ALI mice.

### TRQ Reduced Proinflammatory Mediators in Serum and Lung Tissues of ALI Mice

**FIGURE 4:**
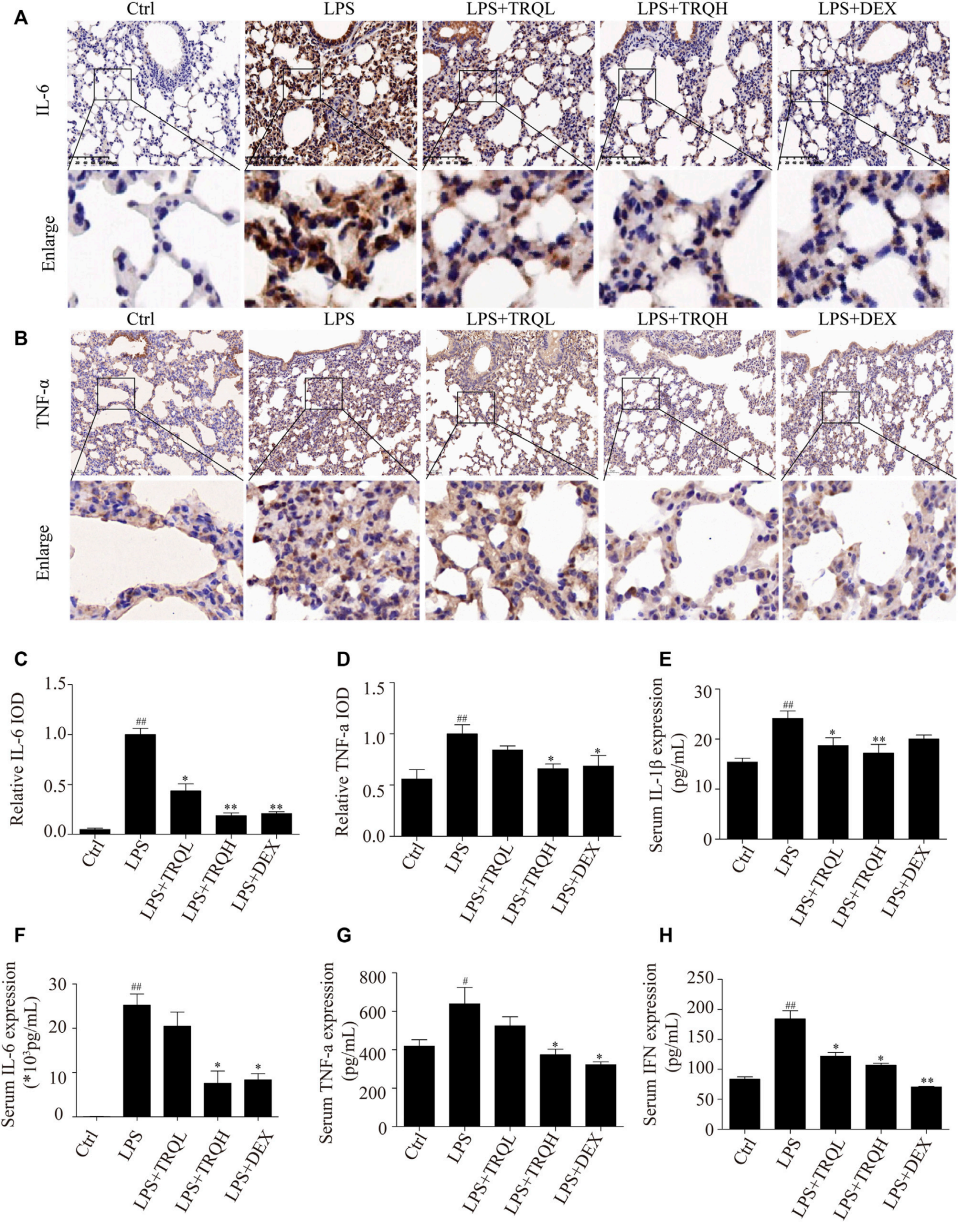
TRQ decreased inflammatory mediator production in LPS-induced ALI mice. **(A–D)** Representative images and quantitative analysis of IL-6 and TNF-α in lung tissues. **(E)** The levels of IL-1β in serum. **(F)** The levels of IL-6 in serum. **(G)** The levels of TNF-α in serum. **(H)** The levels of IFN-β in serum. *n* = 5–6. ^*##*^ *p* < 0.01 vs. Ctrl group; ^*^ *p* < 0.05 and ^**^ *p* < 0.01 vs. LPS group, respectively.

Since TRQ decreased inflammatory cell infiltration, so we detected the effects of TRQ on the expression of proinflammatory molecules. LPS significantly increased TNF-α and IL-6 expression in lung tissues, while TRQ and DEX pretreatment significantly decreased these expressions (Figures 4A–D). In addition, the levels of TNF-α, IL-6, IL-1β, and IFN-β in serum were also detected by ELISA kits. Similarly, LPS markedly elevated TNF-α, IL-6, IL-1β, and IFN-β levels, which were significantly reduced by TRQ and DEX pretreatment (Figures 4E–H). These results further revealed that TRQ reduced inflammatory responses in ALI mice.

### TRQ Alleviated Oxidative Stress in Lung Tissues of ALI Mice

**FIGURE 5:**
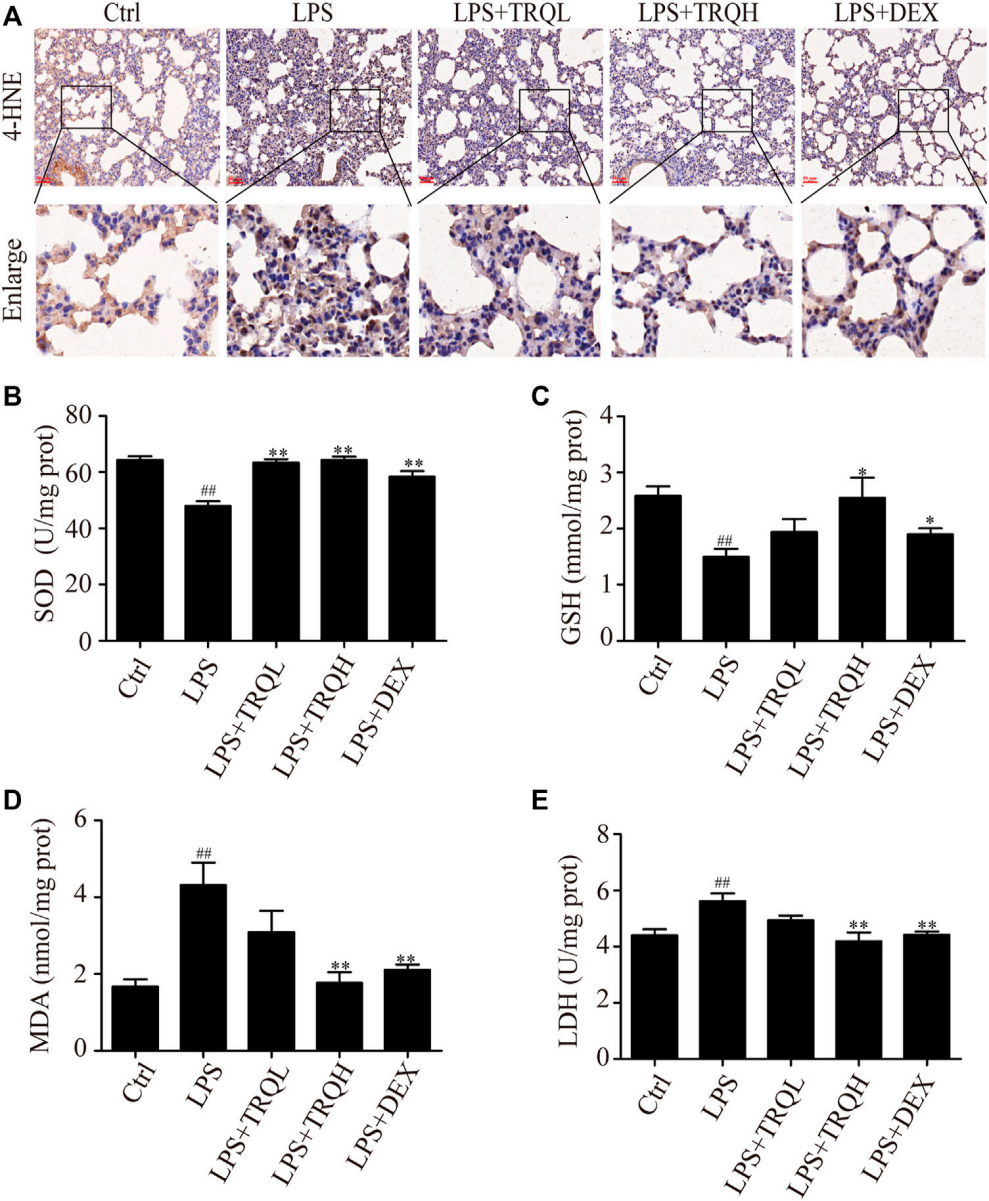
TRQ alleviated oxidative stress in LPS-induced ALI mice. **(A)** The expression of 4-HNE in lung tissues. **(B)** SOD activity in lung tissues. **(C)** GSH activity in lung tissues. **(D)** MDA activity in lung tissues. **(E)** LDH activity in lung tissues. *n* = 5–6. ^*##*^ *p* < 0.01 vs. Ctrl group; ^*^ *p* < 0.05 and ^**^ *p* < 0.01 vs. LPS group, respectively.

Next, we investigated the effects of TRQ on oxidative stress in LPS-induced ALI mice. LPS significantly promoted 4-HNE localization in lung tissues; however, the increased 4-HNE was markedly reduced by TRQ and DEX pretreatment (Figure 5A). Then, the SOD, GSH, MDA, and LDH activities in lung tissues were also detected. LPS remarkably decreased SOD and GSH activities and increased MDA and LDH activities. In contrast, TRQ and DEX treatment significantly increased LPS-suppressed SOD and GSH levels and decreased LPS-increased MDA and LDH contents (Figures 5B–E). Taken together, we conclude that TRQ could suppress oxidative stress in LPS-induced ALI mice.

### TRQ Suppressed LPS-Activated STING Signaling Pathway in ALI Mice

**FIGURE 6:**
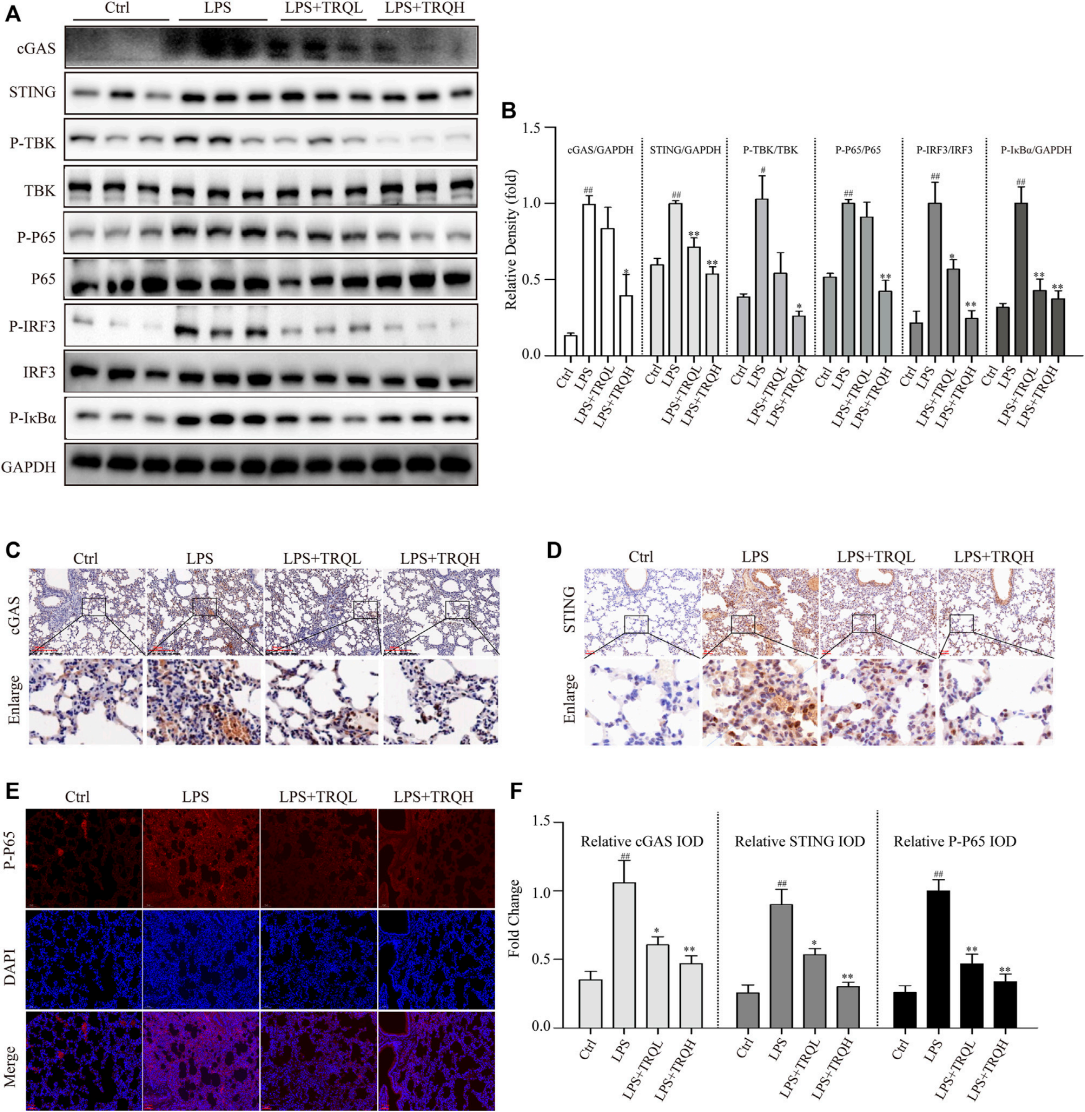
TRQ suppressed STING-mediated IRF3/NF-κB signaling pathways in LPS-induced ALI mice. **(A–B)** The representative images and quantitative analysis of cGAS, STING, P-TBK, TBK, P-P65, P65, P-IRF3, IRF3, and P-IκBα in lung tissues. *n* = 3. **(C–F)** Representative images and quantitative analysis of cGAS, STING, and P-P65 in lung tissues. *n* = 5. ^*##*^ *p* < 0.01 vs. Ctrl group; ^*^ *p* < 0.05 and ^**^ *p* < 0.01 vs. LPS group, respectively.

Given that the STING signaling pathway plays a critical role in regulating inflammatory responses, we thus explored whether it is involved in the therapeutic effects of TRQ in LPS-induced ALI. Both the Western blot and IF staining experiments showed that LPS significantly increased the expression of cGAS and STING and promoted the phosphorylation of TBK, IRF3, P65, and IκBα in lung tissues of ALI mice, suggesting that LPS could activate STING-mediated IRF3/NF-κB signaling pathway (Figures 6A–F). Comparatively, TRQ treatment remarkably blocked cGAS, STING, P-TBK, P-IRF3, P-P65, and P-IκBα production (Figures 6A–F). Meanwhile, TRQ also inhibited the phosphorylation of STING (Supplementary Figure S1A). These results indicated that the therapeutic effects of TRQ on LPS-induced ALI might be related to its inhibition of the STING-mediated IRF3/NF-κB signaling pathway.

### TRQ Inhibited LPS-Induced Inflammatory Cytokines, ROS, and mtDNA Release in RAW 264.7 Cells

**FIGURE 7:**
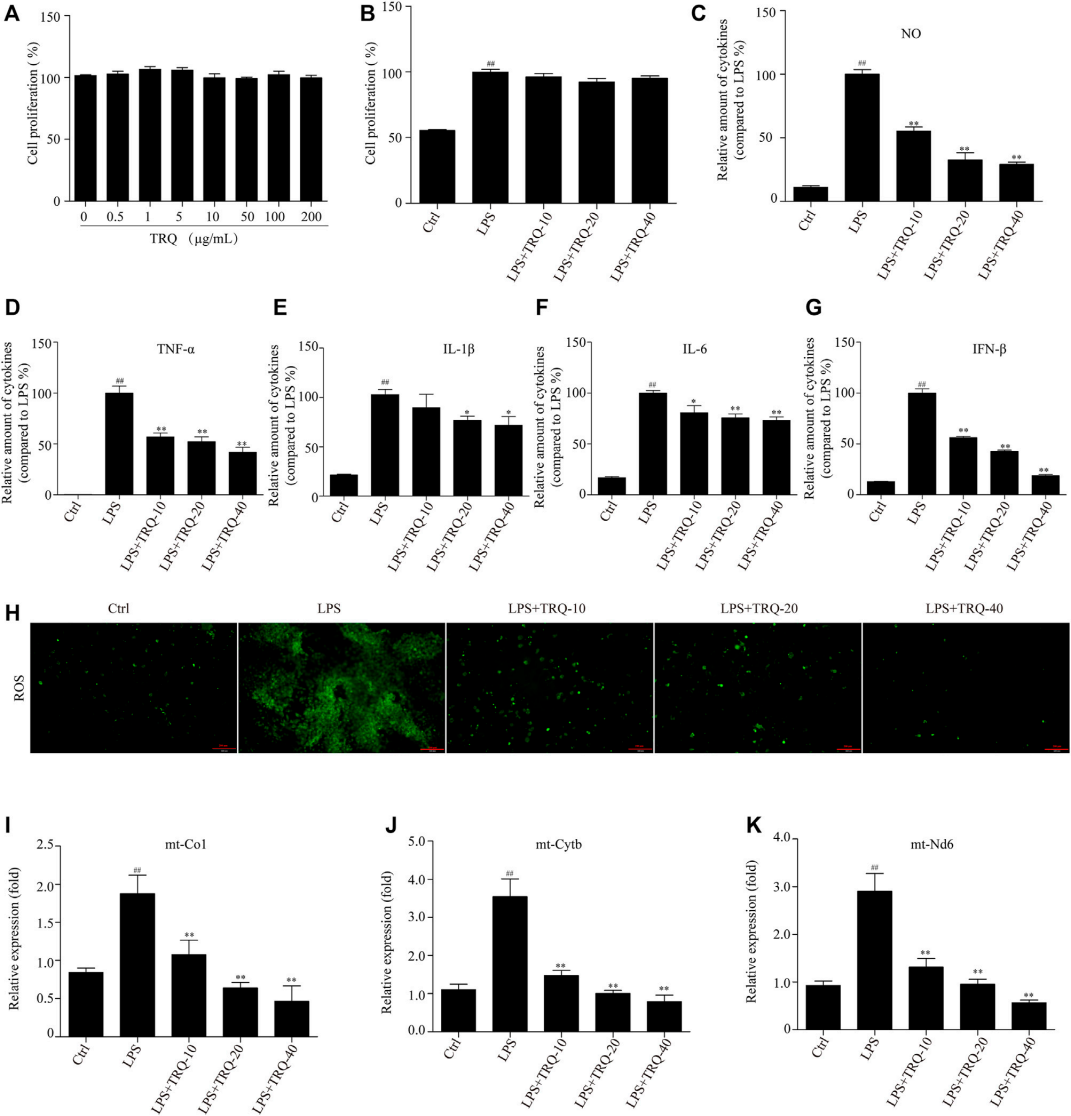
TRQ suppressed inflammatory responses, ROS production, and mtDNA release in LPS-stimulated RAW 264.7 cells. **(A)** The cell proliferation detected by CCK-8. *n* = 3. **(B)** The cell proliferation detected by CCK-8 in the presence of LPS. *n* = 5. **(C–G)** The production of NO, TNF-α, IL-1β, IL-6, and IFN-β in LPS-stimulated RAW 264.7 cells. *n* = 6. **(H)** The ROS production was detected by staining with DCFH-DA. *n* = 5. **(I)** qRT-PCR analysis showing expression of mtDNA. *n* = 4. ^*##*^ *p* < 0.01 vs. Ctrl group; ^*^ *p* < 0.05 and ^**^ *p* < 0.01 vs. LPS group, respectively.

Due to these results *in vivo*, we further wanted to investigate the effects of TRQ in LPS-stimulated RAW 264.7 cells and neutrophils. Firstly, we performed a CCK-8 assay to detect the effects of a series of concentrations of TRQ in or out of the presence of LPS on cell proliferation. The results showed that TRQ at dose up to 200 μg/ml showed no cytotoxicity on RAW 264.7 cells (Figure 7A). LPS significantly promoted cell proliferation, while TRQ (10, 20, and 40 μg/ml) showed no significant effects on the proliferation of LPS-stimulated cells (Figure 7B). LPS treatment significantly upregulated the expression of NO, TNF-α, IL-1β, IL-6, and IFN-β, whereas TRQ considerably downregulated LPS-induced expression of NO, TNF-α, IL-1β, IL-6, and IFN-β in a dose-dependent manner (Figures 7C–G). Moreover, TRQ showed inhibitory effects on the MPO activity in LPS-stimulated neutrophils (Supplementary Figure S2A-B). These results confirmed that TRQ could suppress LPS-induced inflammation. Next, we also checked the production of ROS and cytoplasmic mtDNA, which could activate the STING signaling pathway and promote the production of IFN and other inflammatory cytokines such as TNF-α, IL-1β, and IL-6. TRQ markedly decreased LPS-induced ROS production (Figure 7H). LPS significantly promoted the cytoplasmic mtDNA (mt-*Co1*, *mt-Nd6*, and *mt-Cytb*) expression, while TRQ obviously inhibited mtDNA production, and TRQ at 40 μg/ml reduced the LPS-induced mtDNA release by >60% (Figures 7I–K). These results indicated that TRQ could prevent inflammatory responses, oxidative responses, and mtDNA release *in vitro*.

### TRQ Regulated the STING-Mediated Signaling Pathway in RAW 264.7 Cells

**FIGURE 8:**
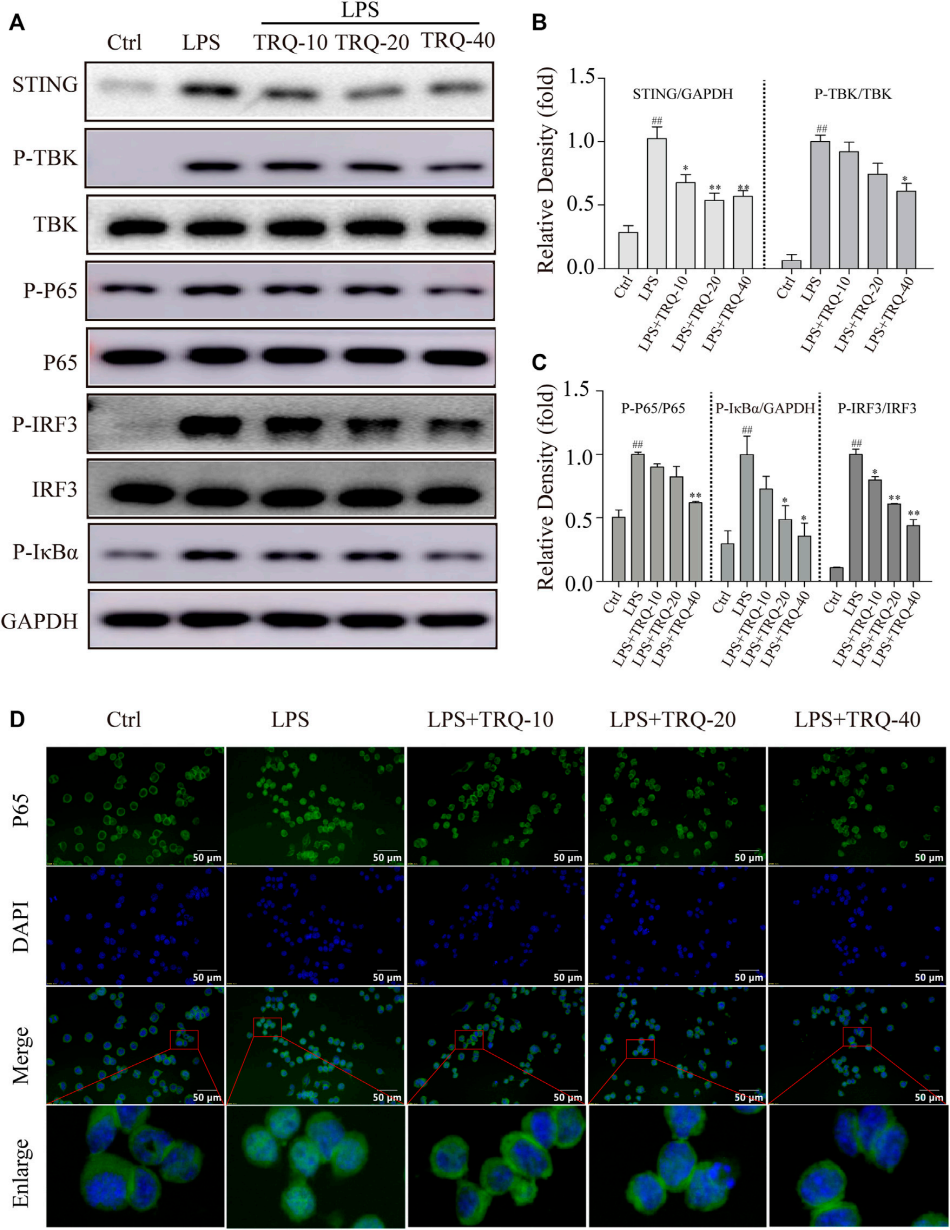
TRQ suppressed STING-mediated IRF3/NF-κB signaling pathway in LPS-stimulated RAW 264.7 cells. **(A–C)** The representative Western blotting measurement of STING, P-TBK, TBK, P-P65, P65, P-IRF3, IRF3, and P-IκBα in LPS-stimulated RAW 264.7 cells. *n* = 3. **(D)** The representative images of IF staining of P65. *n* = 5. ^##^ *p* < 0.01 vs. Ctrl group; ^*^ *p* < 0.05 and ^**^ *p* < 0.01 vs. LPS group, respectively.

Next, we explored whether TRQ could downregulate the STING-mediated IRF3/NF-κB signaling pathway in LPS-stimulated RAW 264.7 cells. LPS promoted STING expression and the phosphorylation of STING, TBK, IRF3, p65, and IκBα while TRQ treatment significantly alleviated these changes (Figures 8A–C) (Supplementary Figure S1B). What is more, the IF staining results showed that LPS increased the nuclear translocation of p65, whereas TRQ suppressed the p65 nuclear translocation induced by LPS in RAW 264.7 cells (Figure 8D). The data confirmed that TRQ could downregulate STING-mediated IRF3/NF-κB signaling pathway.

### TRQ Alleviated ALI Through Regulating STING Signaling Pathway

**FIGURE 9:**
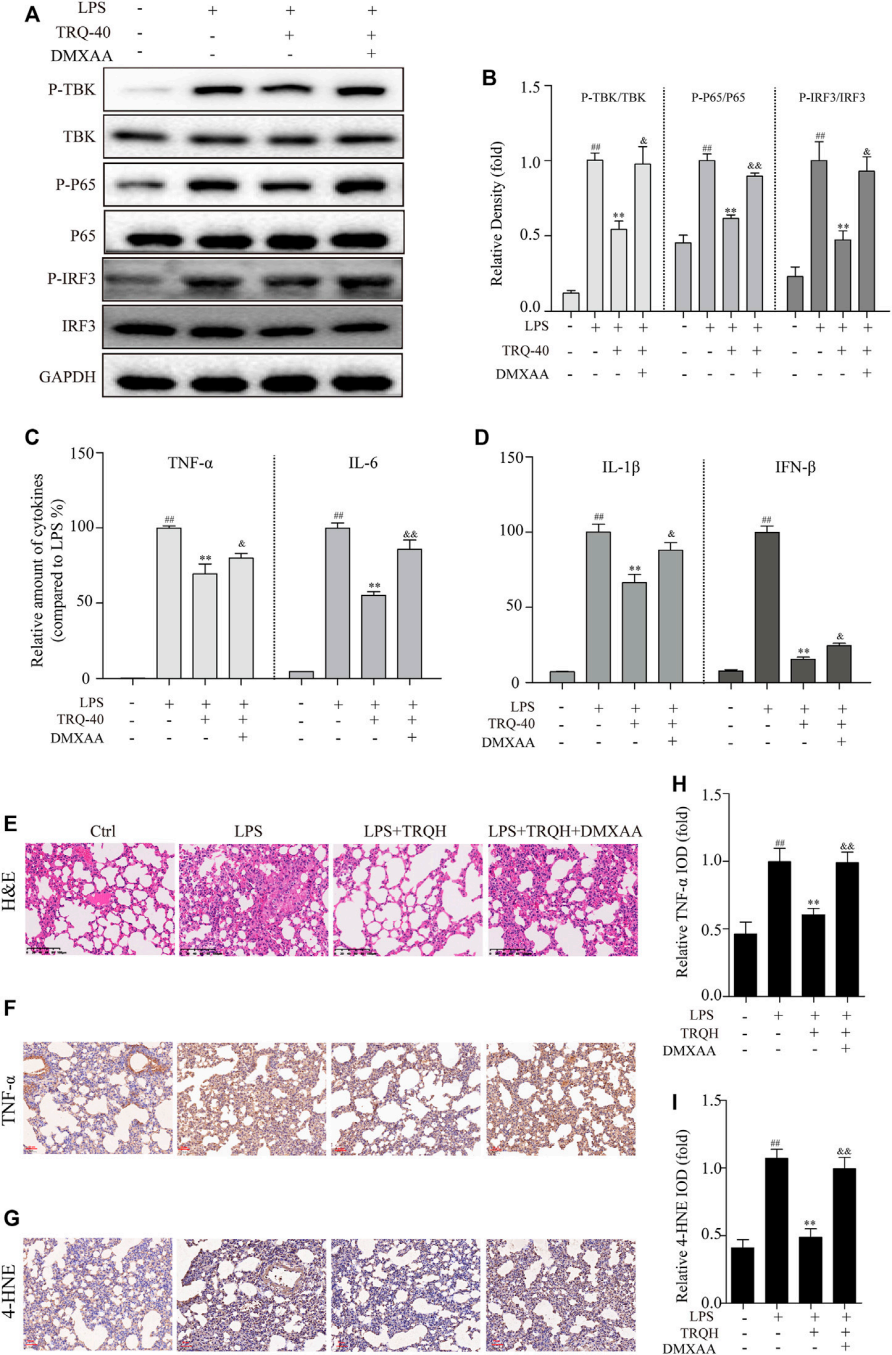
TRQ might prevent acute lung injury (ALI) by suppressing STING signaling pathway. **(A–B)** The representative Western blotting measurement of P-TBK, TBK, P-P65, P65, P-IRF3, and IRF3 in RAW 264.7 cells. *n* = 3. **(C–D)** The expression of TNF-α, IL-6, IL-1β, and IFN-β in RAW 264.7 cells. *n* = 5. **(E)** Representative images of H&E staining in lung tissues. *n* = 5. **(F–I)** The representative images and quantitative analysis of TNF-α and 4-HNE in lung tissues. *n* = 5. ^*##*^ *p* < 0.01 vs. Ctrl group; ^**^ *p* < 0.01 vs. LPS group; ^&^ *p* < 0.05 and ^&&^ *p* < 0.01 vs. LPS + TRQH group, respectively.

In order to investigate whether the protective effects of TRQ were eliminated when STING was activated, we further detected it with DMXAA in LPS-stimulated RAW264.7 cells and ALI mice. TRQ markedly inhibited the activation of the STING-mediated IRF3/NF-κB signaling pathway in LPS-stimulated RAW264.7 cells (Figures 9A,B). However, DMXAA treatment could partially abolish the effects of TRQ and up-regulate STING-mediated IRF3/NF-κB signaling pathway. Meanwhile, DMXXA treatment also partially decreased the inhibition of TRQ on TNF-α, IL-6, IL-1β, and IFN-β production (Figures 9C,D). In addition, the inhibitory effects of TRQ on LPS-induced lung histological changes, TNF-α, and 4-HNE expression were almost abolished with DMXAA in ALI mice (Figures 9E–I). The results indicated that TRQ might prevent LPS-induced inflammatory responses and oxidative stress and alleviate LPS-induced ALI by blocking STING-mediated IRF3/NF-κB signaling pathway.

## Discussion

TRQ injection, a Chinese patent medicine, is clinically used for acute pneumonia and acute bronchitis and recommended in the therapeutic regimens of COVID-19 in China (He et al., 2021). However, the effects and precise mechanism of TRQ on ALI are still unknown. In this work, we detected the protective effects of TRQ on LPS-induced ALI and explored the underlying mechanism. The results showed that TRQ protected against lung edema, lung injury, and inflammatory cell infiltration, alleviated inflammatory responses and oxidative stress, and inactivated STING-mediated IRF3/NF-κB signaling pathway *in vivo* and *in vitro*. Further, we showed that inhibitory effects of TRQ on inflammatory responses, oxidative stress, lung injury, and STING-mediated IRF3/NF-κB signaling pathway were partly abolished by DMXAA. Accordingly, the evidence supported that TRQ could exert antioxidant and anti-inflammatory activities to prevent LPS-induced ALI involving its downregulation of the STING signaling pathway.

ALI is a life-threatening lung disease, accompanied by acute and serious inflammatory responses. Therefore, LPS was considered as an ideal substance to construct the ALI model (Chen et al., 2019). In LPS-induced ALI, numerous pieces of evidence showed that lung pathological change, lung edema, and increased pulmonary permeability are involved in the progress of ALI (Yao et al., 2014). Previous studies showed that TRQ could effectively attenuate airway inflammation by decreasing inflammatory responses in the LPS-induced rats model (Liu et al., 2016; Liu et al., 2020). However, its effect on ALI was rarely reported. In this study, we found that TRQ treatment effectively improved LPS-induced lung pathological change and decreased lung edema and inflammatory cells accumulation in lung tissues of LPS-induced ALI. Lung W/D ratio and BALF protein levels were considered as measurements of lung edema and pulmonary permeability (Ma et al., 2021). Our findings showed that TRQ decreased LPS-increased lung W/D ratio and total protein concentration in BALF. Thus, TRQ could improve LPS-induced lung edema and pulmonary permeability.

The acute and uncontrolled inflammatory response was the prominent feature in LPS-induced ALI. In LPS-induced ALI mice, the overaccumulation of inflammatory cells (neutrophils and macrophages) in lung could promote the production of proinflammatory cytokines, leading to a severer condition (Lei et al., 2020). TRQ significantly decreased the number of inflammatory cells (neutrophils and macrophages) in lung and reduced MPO activity which was produced by neutrophils. The inflammatory mediators, including TNF-α, IL-1β, IL-6, and IFN-β, are closely associated with the exaggeration of ALI (Wang M. et al., 2021). Our results showed that TRQ remarkably reduced TNF-α, IL-1β, IL-6, and IFN-β production in BALF of ALI mice and LPS-stimulated RAW 264.7 cells. Taken together, TRQ could mitigate inflammation in LPS-induced ALI mice and RAW 264.7 cells. Consistent with our results, Liu et al. reported that TRQ could decrease inflammatory cells number in BALF and proinflammatory cytokines (TNF-α, IL-1β, and IL-6) production in mice with LPS-induced airway inflammation (Liu et al., 2016). The results suggested that TRQ could prevent inflammatory responses *in vivo* and *in vitro*.

During the development of ALI, inflammatory molecules can enhance oxidative stress; in turn, oxidative stress causes more serious inflammatory responses (Cen et al., 2021). Previous studies focused on the anti-inflammatory and antibacterial effects of TRQ, but its effects on oxidative stress remained uninvestigated. In this study, we found that TRQ dramatically alleviated LPS-induced oxidative stress demonstrated by increased SOD and GSH production and decreased 4-HNE, MDA, and LDH content in lung tissues. In addition, TRQ also suppressed ROS production in LPS-stimulated RAW 264.7 cells. Taken together, these data indicated that TRQ could prevent LPS-induced ALI by mitigating inflammatory responses and oxidative stress.

Since the TRQ has a definite effect on LPS-induced ALI, we further explore the underlying mechanism. STING as a cytoplasmic DNA sensor not only promotes IRF activation but also activates the NF-κB signaling pathway, resulting in the expression of IFN and other inflammatory mediators such as IFN, TNF-α, and IL-6 (Balka et al., 2020). Luo et al. reported that the expression of STING was upregulated in the liver of NAFLD patients, and the loss of STING in liver macrophages could alleviate NF-κB and JNK1 activation, relieve inflammatory responses, and prevent the severity of liver fibrosis in mice with NAFLD (Luo et al., 2018). Sun et al. found that RTA-408 (Nrf2 activator) could inhibit osteoclastogenesis and prevent bone loss in ovariectomy-induced osteoporosis mice models *via* blocking STING dependent NF-κB signaling pathway (Sun et al., 2020). However, whether STING-mediated IRF3/NF-κB signaling pathway is involved in LPS-induced ALI is unknown. In this study, we found that LPS could promote mtDNA release, elevate STING expression, and increase TBK, IRF3, NF-κB, and IκBα phosphorylation, suggesting that STING-mediated IRF3/NF-κB was involved in LPS-induced ALI. Consistent with the results in our study, Li et al. revealed that LPS could promote mtDNA production and STING expression in macrophages and lung, and the loss of STING could alleviate LPS-induced lung injury (Ning et al., 2020). Further, we explored whether STING-mediated IRF3/NF-κB signaling pathway was involved in the protective effect of TRQ on LPS-induced ALI. The results led to the conclusion that TRQ significantly inhibited cytoplasmic mtDNA release and downregulated STING-mediated IRF3/NF-κB signaling pathway in LPS-induced ALI mice and RAW 264.7 cells. To further clarify the role of STING signaling pathway in the protective effects of TRQ on LPS-induced ALI, we used the specific STING agonist DMXAA. As expected, DMXAA treatment significantly diminished the inhibition effects of TRQ on STING-mediated IRF3/NF-κB signaling pathway and reversed anti-inflammatory effects of TRQ *in vitro*. In addition, DMXAA treatment also abolished the protection of TRQ against ALI in mice models. Collectively, these data indicated that TRQ could inhibit LPS-induced ALI through downregulating STING-mediated IRF3/NF-κB signaling pathway.

## Conclusion

In conclusion, the findings indicated that TRQ can effectively protect against LPS-induced ALI by inhibiting oxidative stress and inflammatory responses, which might be associated with interfering STING signaling pathway. This study might provide new insight into TRQ for the treatment of respiratory-related diseases. However, there are some limitations to this work. First, the effects of TRQ pretreatment in LPS-induced ALI mice were detected, while the effects of TRQ posttreatment were not determined. Second, we just explored the anti-ALI activities of TRQ injection; however, we have not determined the potential constituents in TRQ injection that exerted the major protective effects. Further experiments are needed to explore the effects of TRQ posttreatment in LPS-induced ALI mice and confirm the potential constituents in TRQ injection that possesses the major anti-ALI activities.

## Data Availability

The raw data supporting the conclusion of this article will be made available by the authors, without undue reservation.
