# Supplementary material for: Tanreqing Inhibits LPS-Induced Acute Lung Injury In Vivo and In Vitro Through Downregulating STING Signaling Pathway
Source: Front Pharmacol. 2021 Oct 14;12:746964. doi: 10.3389/fphar.2021.746964 (PMC8552121; doi:10.3389/fphar.2021.746964)
Supplement: Supplementary file 1 [file DataSheet2.DOCX]

Supplementary materials 2

**Figure 7 (P-P65 and P65)**

**P-P65(n=3)**


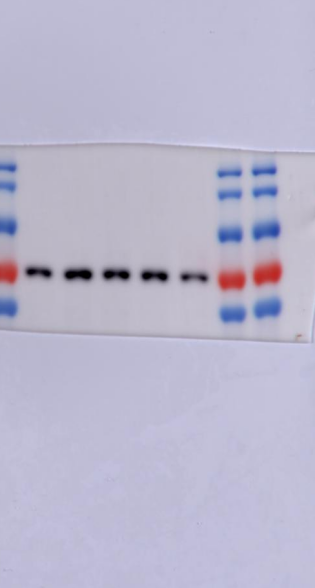

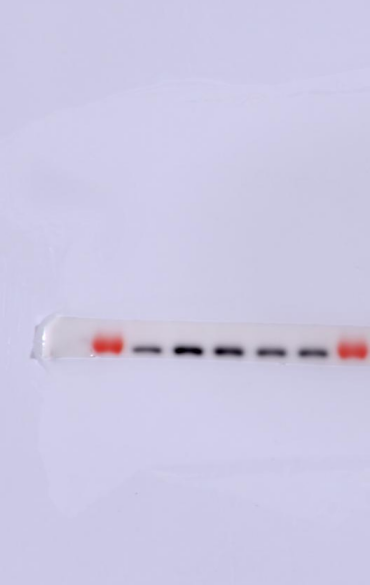

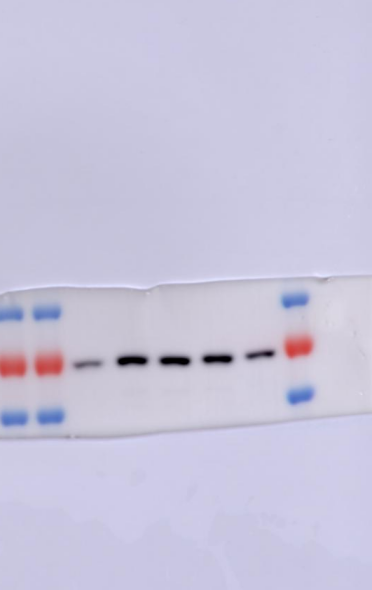


**5**

**2**

**5**

**4**

**3**

**1**

**4**

**3**

**2**

**1**

**5**

**4**

**3**

**2**

**1**

**P65(n=3)**


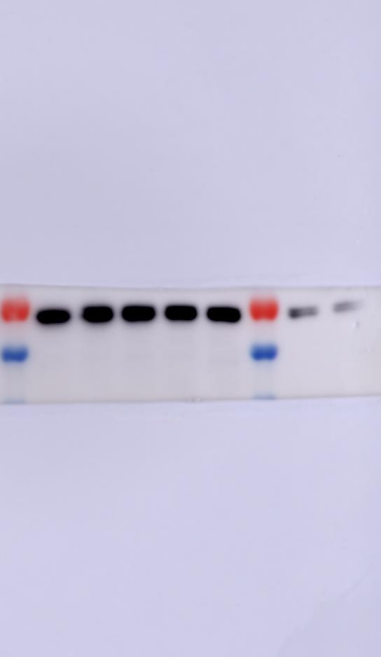

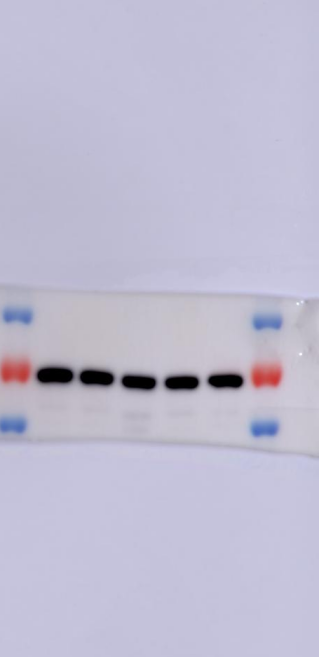

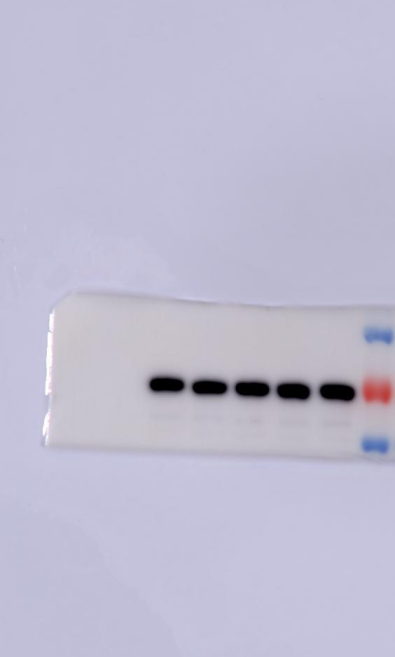


**5**

**4**

**3**

**2**

**1**

**5**

**4**

**3**

**2**

**1**

**1**

**2**

**3**

**4**

**5**

Table 1 The western blot assay of P-P65/P65 (Repeat 1)

| **Lane** | **Gray value (P-P65)** | **Gray value (P65)** | **P-P65/P65 ratio** | **Fold** |
| --- | --- | --- | --- | --- |
| 1 | 19714.0240 | 33551.5390 | 0.5876 | 0.6043 |
| 2 | 27416.4590 | 29311.0040 | 0.9354 | 0.9620 |
| 3 | 23971.3880 | 28578.0040 | 0.8388 | 0.8627 |
| 4 | 24360.5100 | 27485.1750 | 0.8863 | 0.9116 |
| 5 | 16897.5600 | 28191.4180 | 0.5994 | 0.6165 |

Table 2 The western blot assay of P-P65/P65 (Repeat 2)

| **Lane** | **Gray value (P-P65)** | **Gray value (P65)** | **P-P65/P65 ratio** | **Fold** |
| --- | --- | --- | --- | --- |
| 1 | 14749.0540 | 30098.7310 | 0.4900 | 0.5040 |
| 2 | 28401.5100 | 28627.5890 | 0.9921 | 1.0204 |
| 3 | 25836.4590 | 29707.4890 | 0.8697 | 0.8945 |
| 4 | 19937.9240 | 31145.1250 | 0.6402 | 0.6584 |
| 5 | 19608.3170 | 31657.8320 | 0.6194 | 0.6370 |

Table 3 The western blot assay of P-P65/P65 (Repeat 2)

| **Lane** | **Gray value (P-P65)** | **Gray value (P65)** | **P-P65/P65 ratio** | **Fold** |
| --- | --- | --- | --- | --- |
| 1 | 10686.4180 | 27220.5390 | 0.3926 | 0.4038 |
| 2 | 28904.6810 | 29214.1250 | 0.9894 | 1.0176 |
| 3 | 27897.7820 | 30388.1960 | 0.9180 | 0.9442 |
| 4 | 24653.9240 | 28176.8820 | 0.8750 | 0.8999 |
| 5 | 16767.0750 | 28613.2460 | 0.5860 | 0.6027 |

**Figure 5 (STING, P-IκBα, GAPDH)**

**GAPDH (n=3)**

**
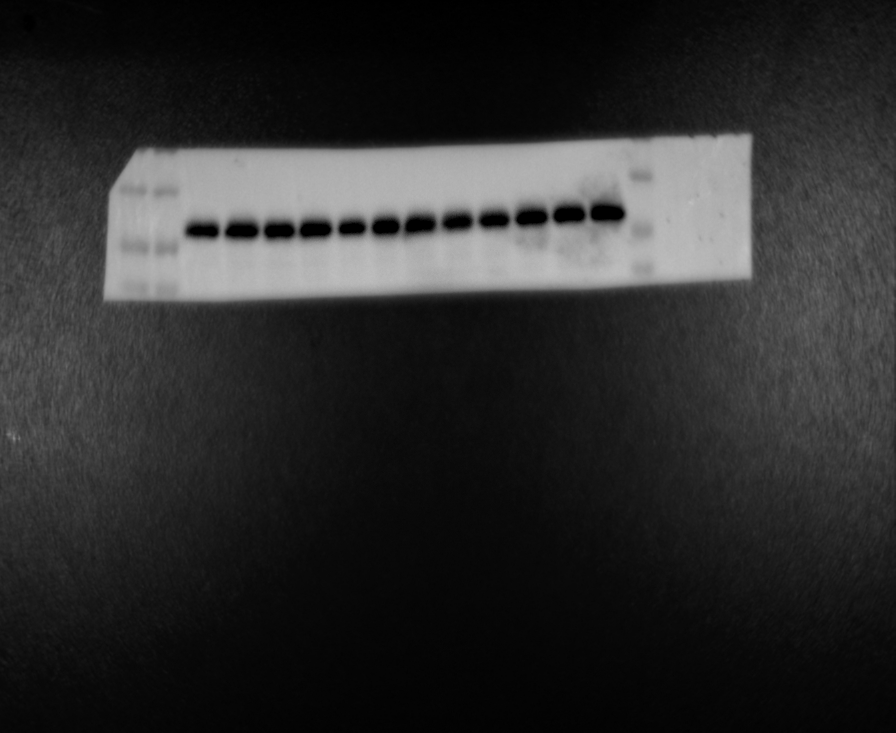
**

**11**

**12**

**10**

**9**

**8**

**6**

**7**

**5**

**4**

**3**

**2**

**1**

**STING(n=3)**

**
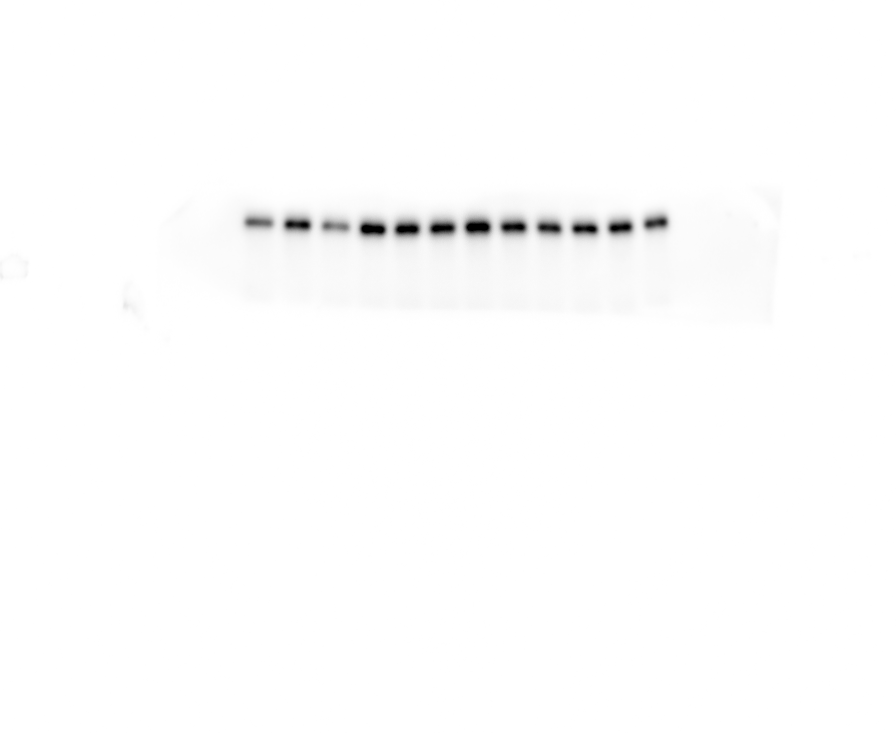
**

**11**

**12**

**10**

**9**

**8**

**7**

**6**

**5**

**4**

**3**

**1**

**2**

**P-IκBα(n=3)**

**
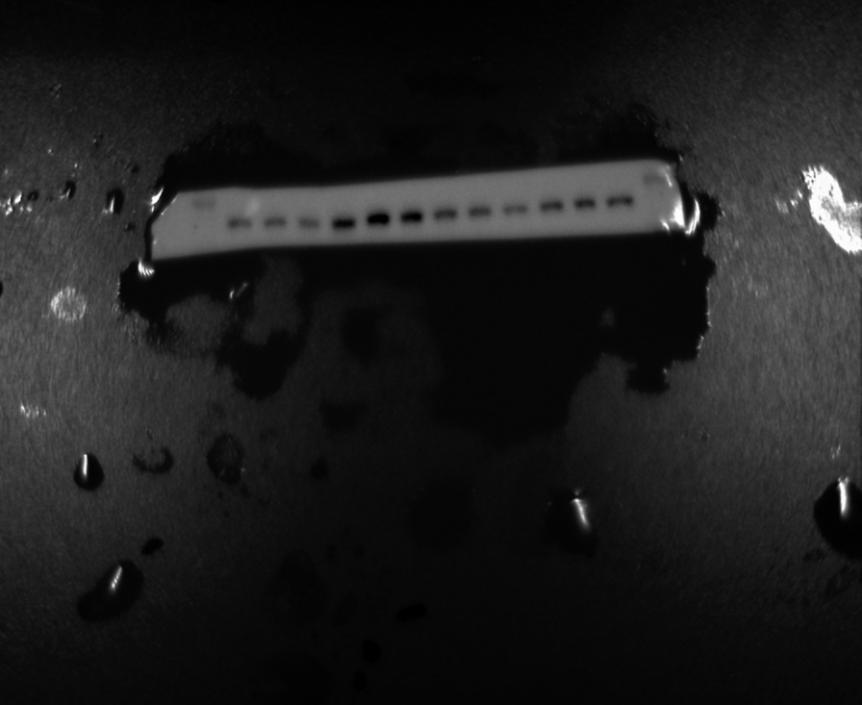
**

**12**

**11**

**10**

**9**

**8**

**7**

**6**

**5**

**4**

**3**

**1**

**2**

Table 4 The western blot assay of STING/GAPDH

|  | **Lane** | **Gray value (STING)** | **Gray value (GAPDH)** | **STING/GAPDH ratio** | **Fold** |
| --- | --- | --- | --- | --- | --- |
| **Ctrl** | 1 | 5841.5480 | 10682.8910 | 0.5468 | 0.5898 |
|  | 2 | 7518.3050 | 12022.5270 | 0.6254 | 0.6745 |
|  | 3 | 5881.4770 | 12022.3050 | 0.4892 | 0.5277 |
| **LPS** | 4 | 10721.9980 | 11900.1130 | 0.9010 | 0.9718 |
|  | 5 | 9208.1840 | 9614.3550 | 0.9578 | 1.0331 |
|  | 6 | 9210.0620 | 9984.8200 | 0.9224 | 0.9949 |
| **LPS+TRQL** | 7 | 10148.0230 | 14570.5980 | 0.6965 | 0.7512 |
|  | 8 | 7985.5980 | 10829.6480 | 0.7374 | 0.7954 |
|  | 9 | 6809.0620 | 12353.1840 | 0.5512 | 0.5945 |
| **LPS+TRQH** | 10 | 7410.8910 | 13886.3050 | 0.5337 | 0.5756 |
|  | 11 | 7089.9840 | 12881.2340 | 0.5504 | 0.5937 |
|  | 12 | 6494.8220 | 15835.3050 | 0.4101 | 0.4424 |

Table 5 The western blot assay of P-IκBα/GAPDH

|  | **Lane** | **Gray value (STING)** | **Gray value (GAPDH)** | **STING/GAPDH ratio** | **Fold** |
| --- | --- | --- | --- | --- | --- |
| **Ctrl** | 1 | 4203.6480 | 10682.8910 | 0.3935 | 0.3510 |
|  | 2 | 4538.4770 | 12022.5270 | 0.3775 | 0.3367 |
|  | 3 | 3600.6480 | 12022.3050 | 0.2995 | 0.2672 |
| **LPS** | 4 | 10466.7190 | 11900.1130 | 0.8795 | 0.7846 |
|  | 5 | 12332.3050 | 9614.3550 | 1.2827 | 1.1442 |
|  | 6 | 11990.3550 | 9984.8200 | 1.2009 | 1.0712 |
| **LPS+TRQL** | 7 | 8821.8910 | 14570.5980 | 0.6055 | 0.5401 |
|  | 8 | 5612.0120 | 10829.6480 | 0.5182 | 0.4623 |
|  | 9 | 3882.4770 | 12353.1840 | 0.3143 | 0.2804 |
| **LPS+TRQH** | 10 | 5900.4320 | 13886.3050 | 0.4249 | 0.3790 |
|  | 11 | 6698.0120 | 12881.2340 | 0.5200 | 0.4638 |
|  | 12 | 5002.4210 | 15835.3050 | 0.3159 | 0.2818 |

**Figure 7 (P-TBK, P-IRF3, )**

1. **TBK (n=3)**

**
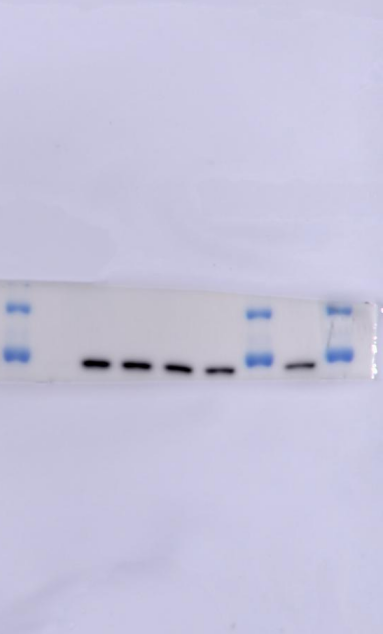

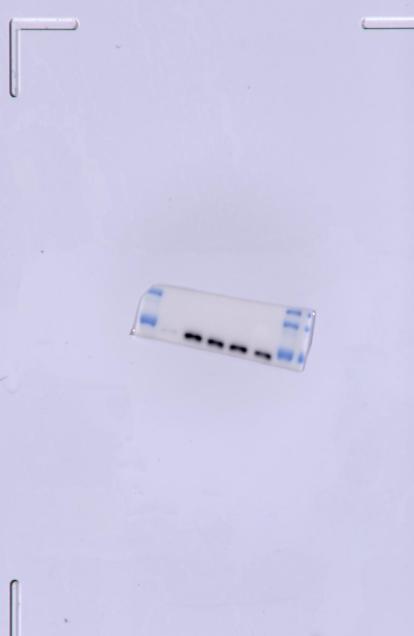

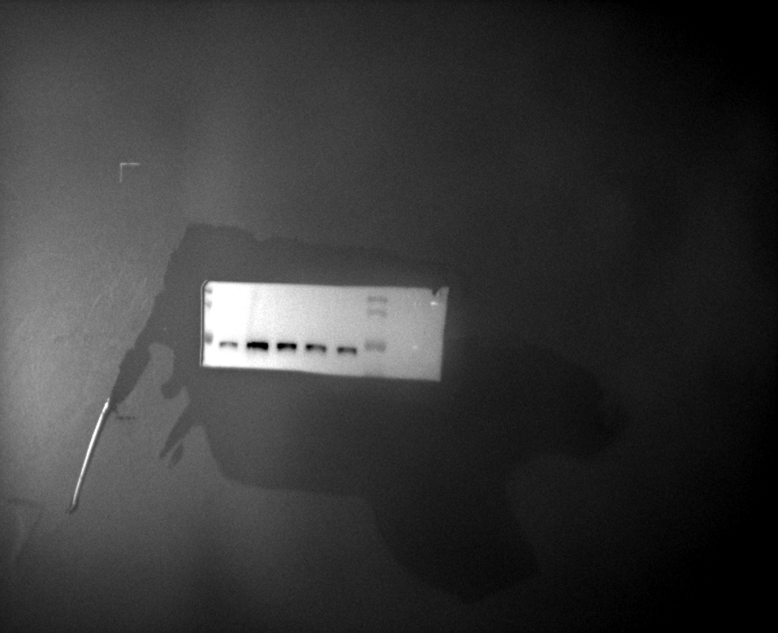
**

**5**

**4**

**3**

**2**

**1**

**5**

**4**

**3**

**1**

**5**

**4**

**3**

**2**

**1**

**2**

**TBK (n=3)**

**
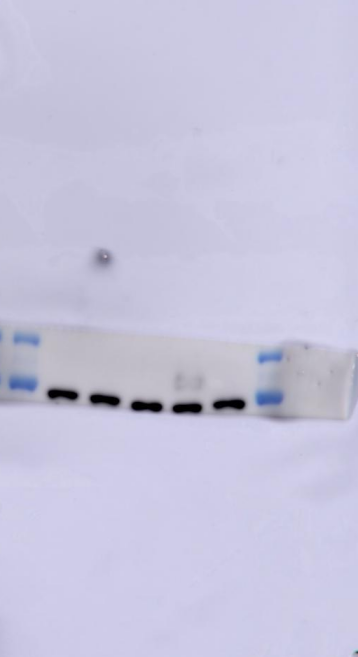

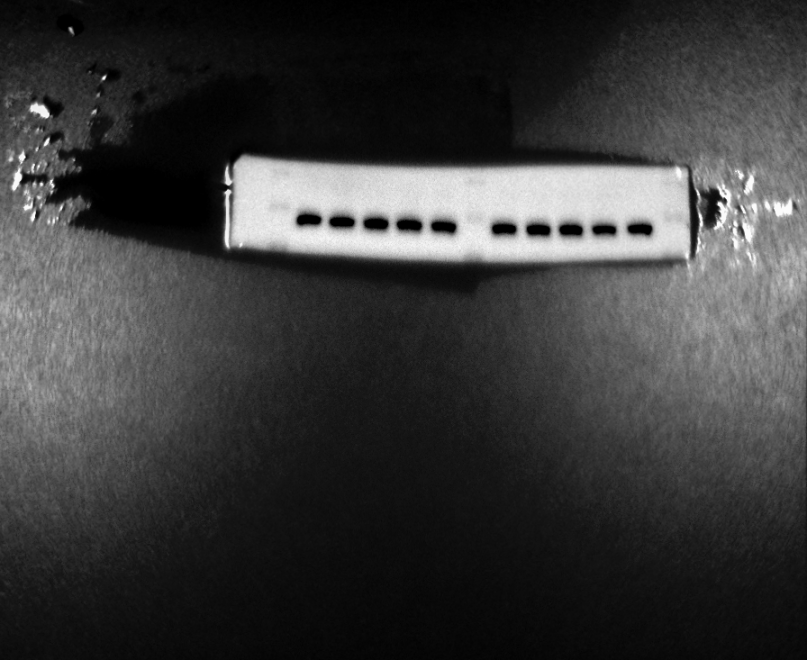
**

**5**

**4**

**3**

**5**

**4**

**3**

**2**

**3**

**4**

**5**

**1**

**2**

**2**

**1**

**1**

Table 6 The western blot assay of P-TBK/TBK (Repeat 1)

| **Lane** | **Gray value (P-TBK)** | **Gray value (TBK)** | **P-TBK/TBK ratio** | **Fold** |
| --- | --- | --- | --- | --- |
| 1 | 397.3350 | 24815.8530 | 0.0160 | 0.0149 |
| 2 | 28019.9740 | 24799.8530 | 1.1298 | 1.0546 |
| 3 | 27457.7310 | 23984.3880 | 1.1448 | 1.0686 |
| 4 | 24451.9530 | 28319.6810 | 0.8634 | 0.8060 |
| 5 | 19434.4390 | 28911.4390 | 0.6722 | 0.6275 |

Table 7 The western blot assay of P-TBK/TBK (Repeat 2)

| **Lane** | **Gray value (P-TBK)** | **Gray value (TBK)** | **P-TBK/TBK ratio** | **Fold** |
| --- | --- | --- | --- | --- |
| 1 | 537.9710 | 27089.4390 | 0.0199 | 0.0185 |
| 2 | 31740.8940 | 28215.9740 | 1.1249 | 1.0501 |
| 3 | 27716.9240 | 29839.0450 | 0.9289 | 0.8671 |
| 4 | 28112.7520 | 30830.6310 | 0.9118 | 0.8512 |
| 5 | 21395.9740 | 28263.1250 | 0.7570 | 0.7066 |

Table 8 The western blot assay of P-TBK/TBK (Repeat 3)

| **Lane** | **Gray value (P-TBK)** | **Gray value (TBK)** | **P-TBK/TBK ratio** | **Fold** |
| --- | --- | --- | --- | --- |
| 1 | 5054.9740 | 29972.8530 | 0.1687 | 0.1574 |
| 2 | 27934.8740 | 29121.1460 | 0.9593 | 0.8954 |
| 3 | 25644.9240 | 28993.1960 | 0.8845 | 0.8256 |
| 4 | 15450.9740 | 25183.4390 | 0.6135 | 0.5727 |
| 5 | 13888.4390 | 26262.3880 | 0.5288 | 0.4936 |

**P-IRF3 (n=3)**

**
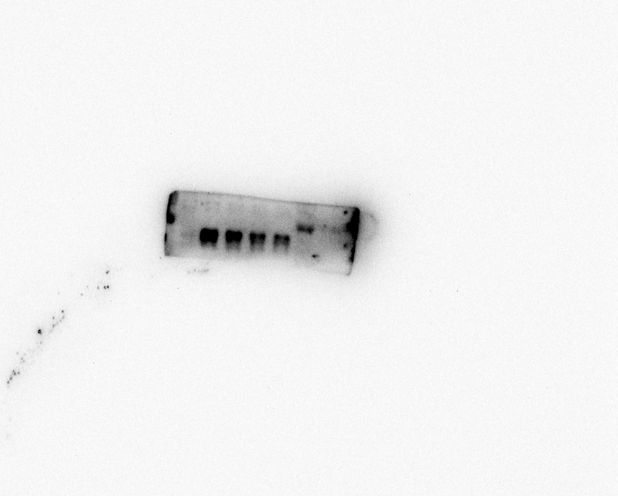

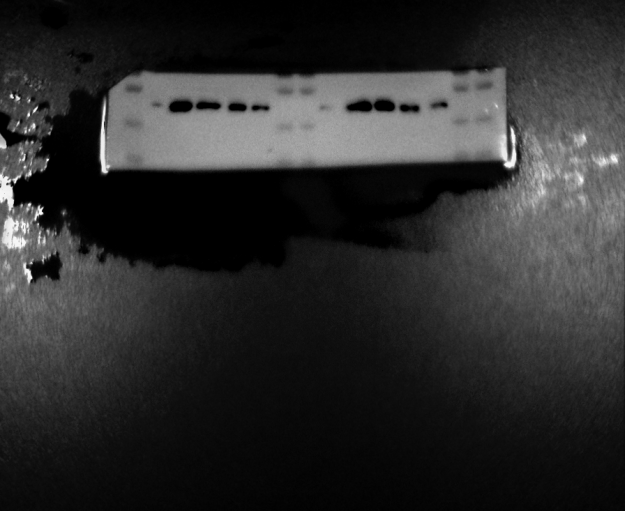
**

**5**

**4**

**3**

**2**

**1**

**5**

**4**

**3**

**2**

**1**

**5**

**4**

**3**

**2**

**1**

**IRF3 (n=3)**

**
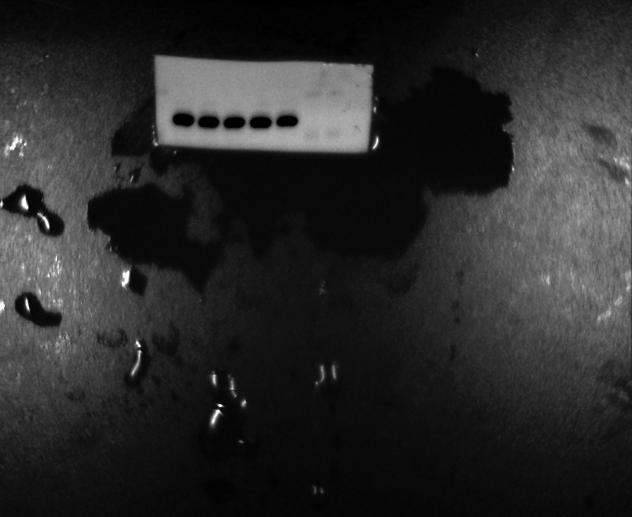

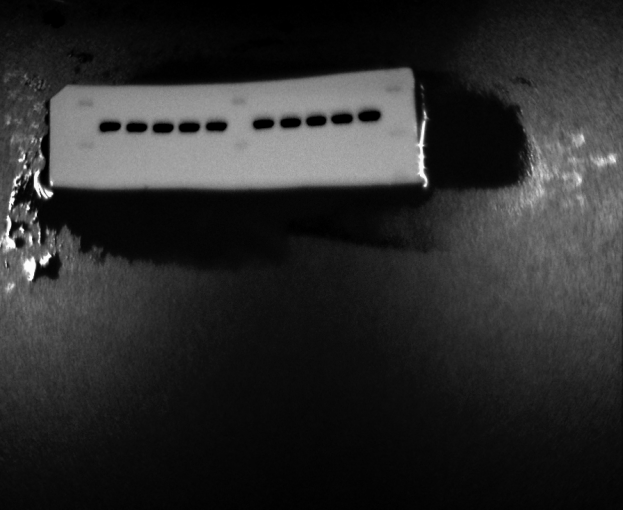
**

**51**

**4**

**3**

**2**

**1**

**1**

**2**

**3**

**4**

**5**

**5**

**4**

**3**

**2**

**1**

Table 8 The western blot assay of P-IRF3/IRF3 (Repeat 1)

| **Lane** | **Gray value (P-IRF3)** | **Gray value (IRF3)** | **P-IRF3/IRF3 ratio** | **Fold** |
| --- | --- | --- | --- | --- |
| 1 | 4237.4680 | 31786.6600 | 0.1333 | 0.1169 |
| 2 | 32856.4590 | 31323.8530 | 1.0489 | 0.9195 |
| 3 | 26765.3380 | 31006.9030 | 0.8632 | 0.7567 |
| 4 | 21346.5100 | 30906.2170 | 0.6907 | 0.6055 |
| 5 | 16717.1960 | 27648.7110 | 0.6046 | 0.5301 |

Table 9 The western blot assay of P-IRF3/IRF3 (Repeat 2)

| **Lane** | **Gray value (P-IRF3)** | **Gray value (IRF3)** | **P-IRF3/IRF3 ratio** | **Fold** |
| --- | --- | --- | --- | --- |
| 1 | 3678.4680 | 29910.9240 | 0.1230 | 0.1078 |
| 2 | 36558.7520 | 30235.9740 | 1.2091 | 1.0600 |
| 3 | 27047.2380 | 29999.6100 | 0.9016 | 0.7904 |
| 4 | 21827.4890 | 30963.5600 | 0.7049 | 0.6180 |
| 5 | 13445.2460 | 32322.6100 | 0.4160 | 0.3647 |

Table 10 The western blot assay of P-IRF3/IRF3 (Repeat 3)

| **Lane** | **Gray value (P-TBK)** | **Gray value (TBK)** | **P-TBK/TBK ratio** | **Fold** |
| --- | --- | --- | --- | --- |
| 1 | 3367.9830 | 29430.5600 | 0.1144 | 0.1003 |
| 2 | 34283.8940 | 29445.8030 | 1.1643 | 1.0207 |
| 3 | 30431.1960 | 31421.3880 | 0.9685 | 0.8490 |
| 4 | 21562.6810 | 31253.1960 | 0.6899 | 0.6048 |
| 5 | 13668.9740 | 28589.0750 | 0.4781 | 0.4191 |

**Figure 8**

**P-IRF3 (n=3)**

**
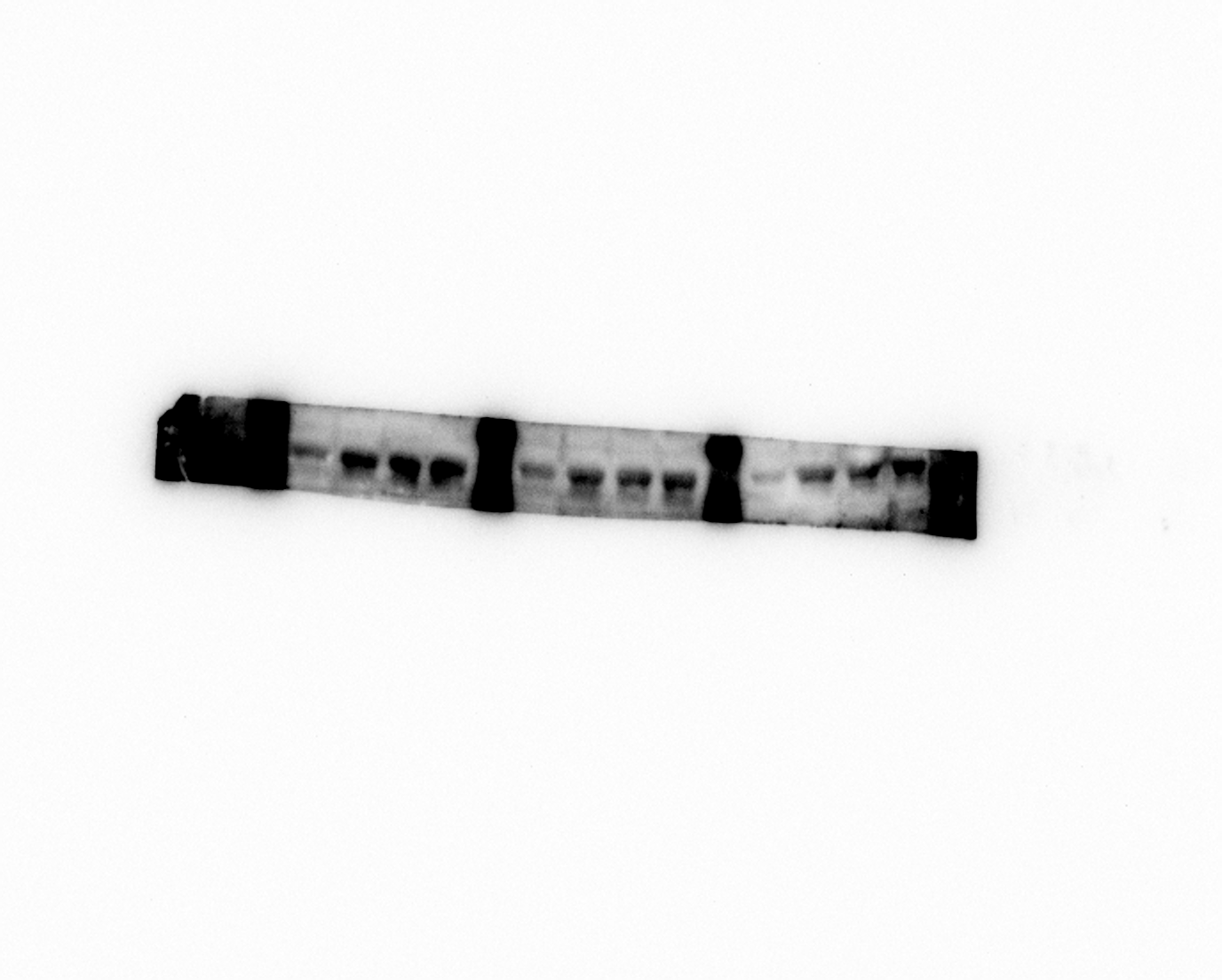

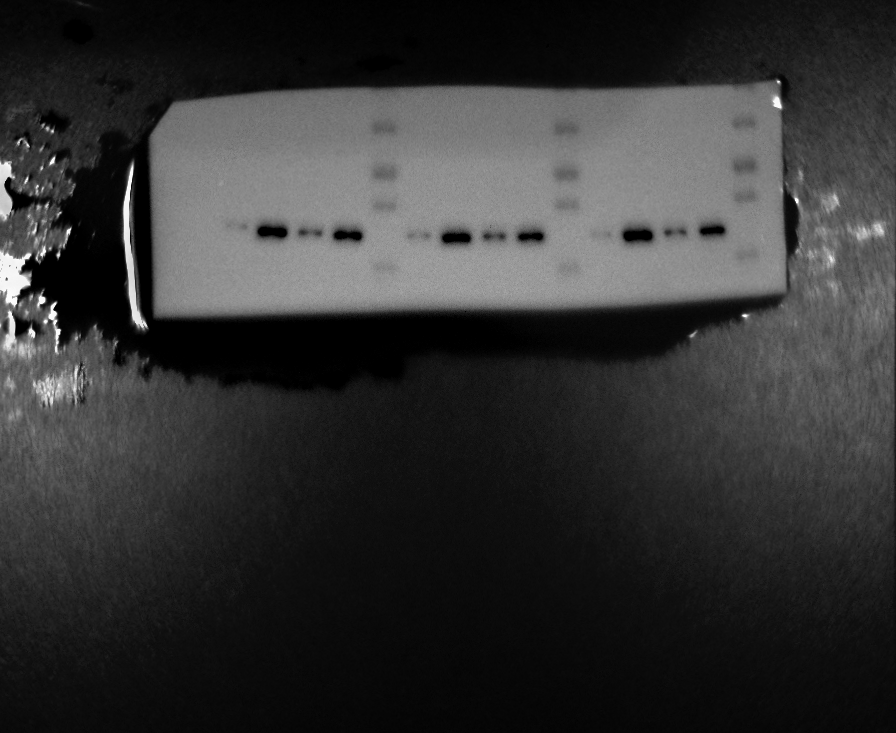

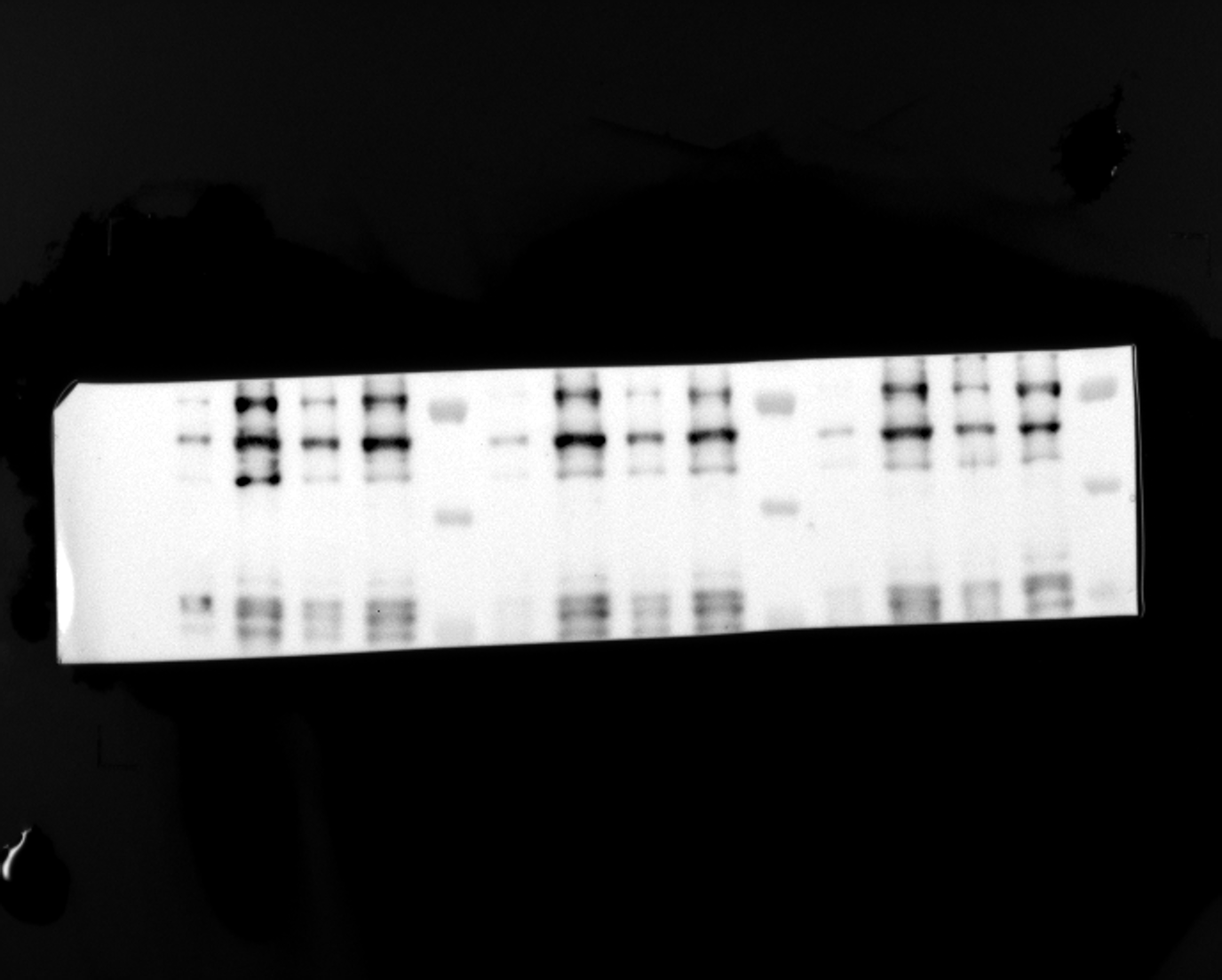
**

**4**

**3**

**1**

**2**

**4**

**3**

**1**

**2**

**2**

**3**

**4**

**1**

**IRF3 (n=3)**

**
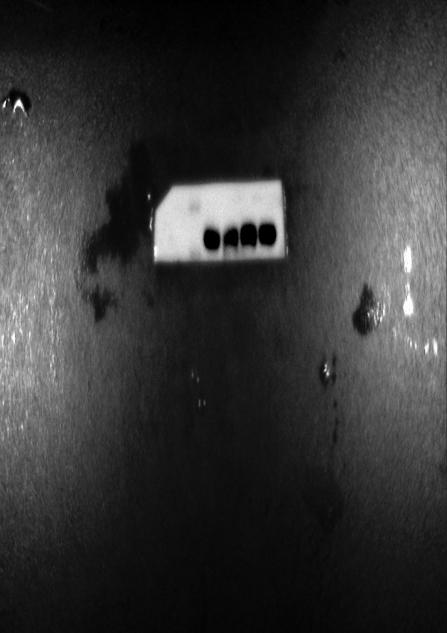

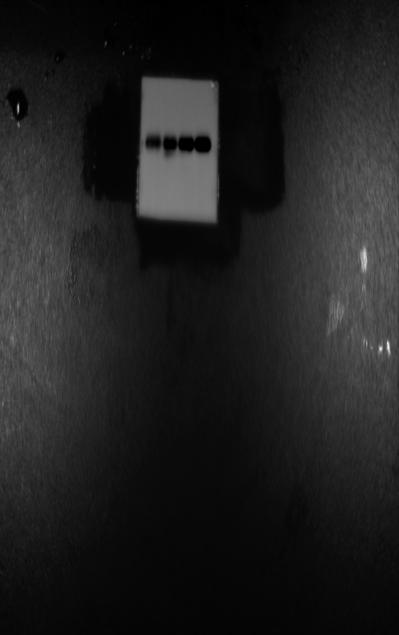

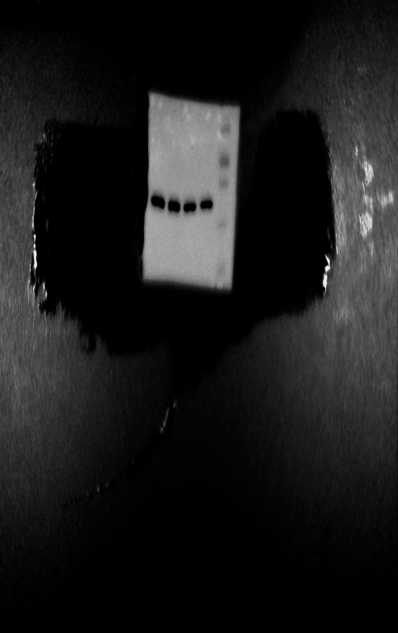
**

**3**

**4**

**2**

**1**

**2**

**4**

**1**

**2**

**3**

**4**

**1**

**3**

Table 11 The western blot assay of P-IRF3/IRF3 (Repeat 1)

| **Lane** | **Gray value (P-IRF3)** | **Gray value (IRF3)** | **P-IRF3/IRF3 ratio** | **Fold** |
| --- | --- | --- | --- | --- |
| 1 | 12235.0750 | 36077.4590 | 0.3391 | 0.3596 |
| 2 | 25393.5300 | 35728.7310 | 0.7107 | 0.7536 |
| 3 | 20058.2880 | 39905.9740 | 0.5026 | 0.5330 |
| 4 | 27457.4590 | 38291.2380 | 0.7171 | 0.7603 |

Table 12The western blot assay of P-IRF3/IRF3 (Repeat 2)

| **Lane** | **Gray value (P-IRF3)** | **Gray value (IRF3)** | **P-IRF3/IRF3 ratio** | **Fold** |
| --- | --- | --- | --- | --- |
| 1 | 4531.3170 | 26785.2880 | 0.1692 | 0.1794 |
| 2 | 36534.3090 | 33126.5100 | 1.1029 | 1.1694 |
| 3 | 12027.1670 | 35781.4890 | 0.3361 | 0.3564 |
| 4 | 33469.5300 | 37893.0240 | 0.8833 | 0.9366 |

Table 13 The western blot assay of P-IRF3/IRF3 (Repeat 3)

| **Lane** | **Gray value (P-IRF3)** | **Gray value (IRF3)** | **P-IRF3/IRF3 ratio** | **Fold** |
| --- | --- | --- | --- | --- |
| 1 | 4290.5180 | 30791.0450 | 0.1393 | 0.1478 |
| 2 | 30030.5720 | 29559.4590 | 1.0159 | 1.0772 |
| 3 | 11883.9950 | 23661.7520 | 0.5022 | 0.5325 |
| 4 | 24465.9950 | 23762.4890 | 1.0296 | 1.0917 |
