# Supplementary material for: Tanreqing Inhibits LPS-Induced Acute Lung Injury In Vivo and In Vitro Through Downregulating STING Signaling Pathway
Source: Front Pharmacol. 2021 Oct 14;12:746964. doi: 10.3389/fphar.2021.746964 (PMC8552121; doi:10.3389/fphar.2021.746964)
Supplement: Supplementary file 2 [file DataSheet1.DOCX]

Supplementary materials 1

**Supplementary Table 1.** Primer sequences for qRT-PCR

| Gene | Forward (5'-3') | Reverse (3'-5') |
| --- | --- | --- |
| *mt-Co1* | GCCCCAGATATAGCATTCCC | GTTCATCCTGTTCCTGCTCC |
| *mt-Cytb* | AGTAGACAAAGCCACCTTGA | CCGCGATAATAAATGGTAAG |
| *mt-Nd6* | TTAGCATTAAAGCCTTCACC | CCAACAAACCCACTAACAAT |
| *18S rDNA* | TAGAGGGACAAGTGGCGTTC | CGCTGAGCCAGTCAGTGT |

**Supplementary Figure 1**


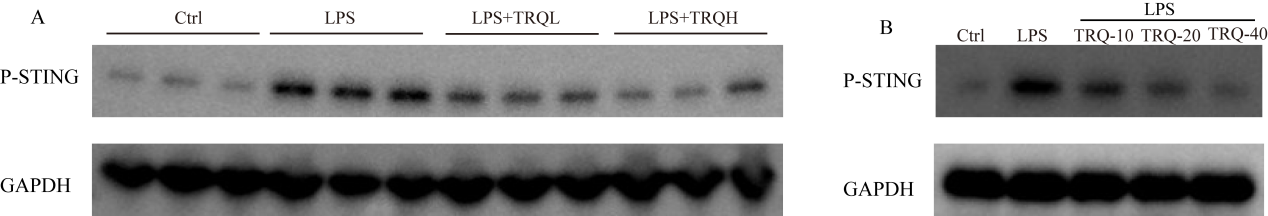


**Supplementary Figure 1.** The effects of TRQ on the phosphorylation of STING. (A) The effects of TRQ on the phosphorylation of STING in lung tissues. (B) The effects of TRQ on the phosphorylation of STING in RAW 264.7 cells.

**Supplementary Figure 2**


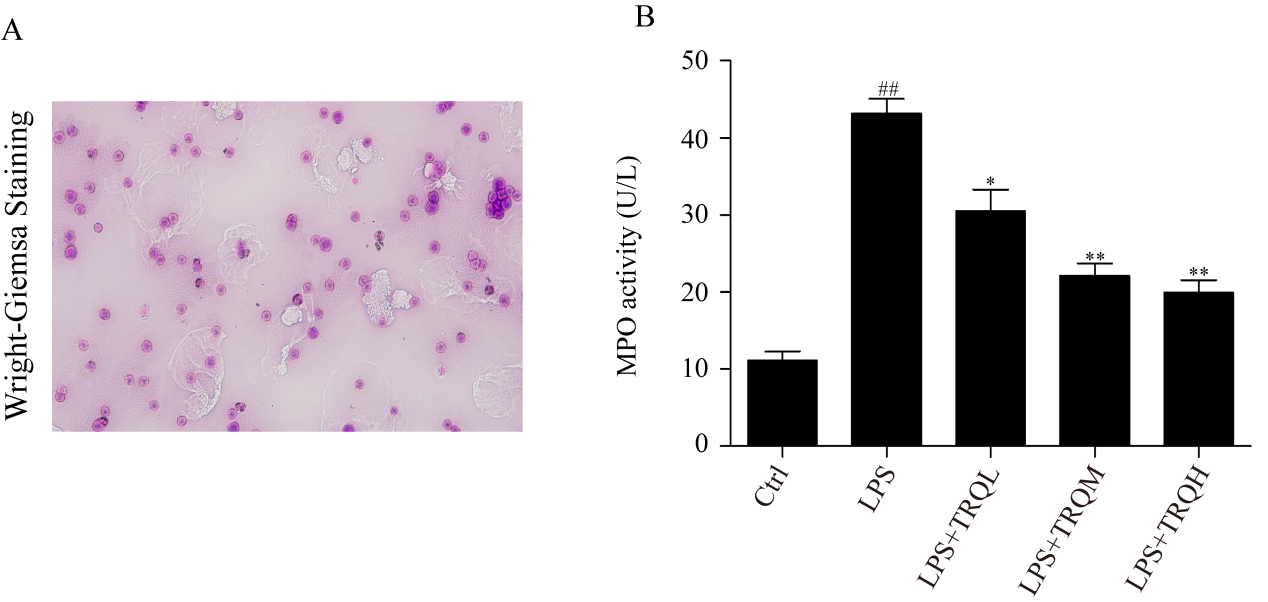


**Supplementary Figure 2.** The effects of TRQ on LPS-stimulated neutrophils. Mouse bone marrow neutrophils was isolated from the femurs. (A) The representative image of neutrophils stained with wright-giemsa solution. (B) The effects of TRQ on the activity of MPO. n=5. ^##^*P* < 0.01 vs. Ctrl group; ^*^*P* < 0.05, ^**^*P* < 0.01 vs. LPS group.

**Supplementary Figure 3**


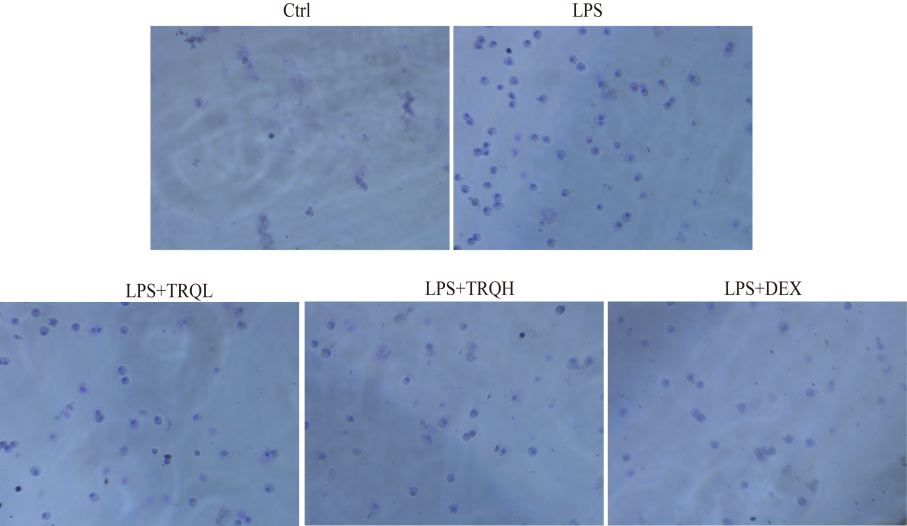


**Supplementary Figure 3.** The effects of TRQ on cell number in the BALF of ALI mice. The cells in the BALF were stained with wright-giemsa solution and photographed under light microscope.
